# Comparative associations of cumulative cholesterol-HDL-glucose index and triglyceride-glucose index with risk of cardiovascular disease in individuals with cardiovascular-kidney-metabolic syndrome stages 0-3: a prospective cohort study

**DOI:** 10.3389/fendo.2026.1950124

**Published:** 2026-09-18

**Authors:** Jiabaili Wang, Qian Liu, Menghui Li, Liming Lin, Yuntao Wu

**Affiliations:** 1 CDUTCM-KEELE Joint Health and Medical Sciences Institute, Chengdu University of Traditional Chinese Medicine, Chengdu, Sichuan, China; 2 Department of Cardiology, Kailuan General Hospital, Tangshan, China; 3 Department of Neurology, Kailuan General Hospital, Tangshan, China

**Keywords:** cardiovascular disease, cardiovascular-kidney-metabolic syndrome, cholesterol, high-density lipoprotein, and glucose index, cumulative, triglyceride-glucose index

## Abstract

**Background:**

The American Heart Association recently introduced Cardiovascular-kidney-metabolic (CKM) syndrome as an integrated clinical construct. Evidence based on a single assessment suggests that the novel composite biomarker, Cholesterol, High-density lipoprotein, and Glucose (CHG) index, is associated with cardiovascular disease (CVD) risk. To data, the association between cumulative CHG index and risk of CVD has not been sufficiently investigated, particularly in individuals with CKM syndrome stages 0-3. Thus, we aimed to investigate the impact of cumulative CHG index on CVD development in individuals with CKM syndrome stages 0–3 and compared the predictive performances of triglyceride-glucose (TyG) index and CHG index for CVD.

**Methods:**

Participants were recruited from the Kailuan Study, a large-scale prospective cohort. The cumulative exposure was assessed using the time-weighted average (TWA) and exposure duration. The optimal cut-off values for TyG_TWA and CHG_TWA associated with CVD were derived using a survival-time method and were used to assess exposure duration of high TyG index/CHG index. Multivariable Cox proportional hazard regression model was used to assess hazard ratio (HR) and 95% confidence interval (95% CI) for CVD incident in individuals with CKM syndrome stages 0-3.

**Results:**

We included 46,827 participants, of these, the mean age was 52.71 years, 76.80% were males. All participants were divided into four groups according to the quartiles (Q) of TyG_TWA and CHG_TWA (Q1-Q4), and exposure duration of high TyG index/CHG index was quantified as 0 (reference), 2, 4, or 6 years. Over a mean follow-up of 11.08 years, 4,639 participants developed CVD. Compared with participants in Q1 group of CHG_TWA, the HRs (95% CIs) of CVD were 1.04 (0.95-1.14) for those in Q2 group, 1.13 (1.04-1.24) for those in Q3 group, and 1.23 (1.12-1.36) for those in Q4 group; compared with participants without exposure of high CHG index, the HR (95% CI) of CVD was increased by 41% (1.25-1.59) for those with 6 years exposure. Similar findings were observed in the analysis of cumulative exposure to TyG index with CVD. The association between TyG_TWA and CVD was linear, whereas the association of CHG_TWA with CVD was non-linear. The result of receiver operating characteristic curve analysis showed that CHG_TWA had a modestly higher discriminatory ability for CVD risk compared with TyG_TWA.

**Conclusions:**

In individuals with CKM syndrome stages 0-3, there was a significant association of high CHG_TWA/TyG_TWA with the risk of CVD, with these associations being stronger with longer exposure duration to high TyG index and CHG index. CHG_TWA demonstrated a modestly higher discriminatory ability for CVD risk compared with TyG_TWA. Our findings suggest that longitudinal monitoring of CHG index and TyG index may provide additional information for CVD risk stratification in individuals with CKM syndrome stages 0-3.

## Introduction

In 2023, the American Heart Association (AHA) defined cardiovascular-kidney-metabolic (CKM) syndrome as a systemic disease arising from pathophysiologic interactions among the cardiovascular system, metabolic risk factors, and chronic kidney disease (CKD) (1, 2). These interactions may substantially increase the risk of multiorgan dysfunction and adverse cardiovascular events (3–6). The accumulation of CKM syndrome components over time will promote the continuous progression of CKM syndrome, thereby increasing the risk of clinical cardiovascular disease (CVD). Evidence from existing studies has established that approximately 90% of individuals are in preclinical stages of CKM syndrome (stages 0-3), around 50% of which are in the progression stages of the disease (7, 8). The AHA emphasized the need to strengthen study on the preclinical stages of CKM syndrome to curb CVD progression and reduce CVD burden. Therefore, the identification of robust biomarkers for the early detection and prevention of CVD has important clinical and public health implications.

Recently, the triglyceride glucose (TyG) index, a surrogate marker of insulin resistance (IR), has been shown to be associated with multiple cardiovascular events (9, 10). In addition, the cholesterol, high density lipoprotein, and glucose (CHG) index, a novel composite biomarker, has demonstrated superior diagnostic performance for type 2 diabetes compared with the TyG index (11). Both the TyG index and CHG index are composite markers that integrate lipid and glucose levels and have been associated with IR and metabolic dysfunction. Although numerous studies have investigated the impact of the two indices on CVD risk, whether CHG index provides additional predictive performance compared with TyG index remains unclear (12). Moreover, some previous studies relied on a single measurement and did not account for their fluctuation over time. Average exposure intensity and exposure duration can comprehensively reflect long-term exposure patterns and cumulative metabolic burden. These approaches have been widely applied in prospective cohort studies (13, 14). Monitoring longitudinal changes in the TyG index and CHG index and investigating their associations with CVD risk may provide additional insights into the role of long-term metabolic exposure in cardiovascular risk stratification. To our knowledge, few studies have comprehensively assessed the associations of cumulative exposure to TyG index and CHG index with CVD risk in individuals with CKM syndrome stages 0–3 or compared their predictive performance for CVD risk.

Therefore, in a large prospective cohort, we investigated the associations of cumulative exposure to TyG index and CHG index with CVD risk among populations with CKM syndrome stages 0-3, and compared their predictive performance for CVD.

## Methods

### Study participants

The data were derived from the Kailuan Study, conducted in Tangshan city of China. All population of the Kailuan group were invited to undergo the health examination conducted every two years. Briefly, 101,510 participants were enrolled in the first health examination conducted between June 2006 and October 2007 (2006 survey). The survey contents included questionnaire, clinical test, and laboratory test at the Kailuan General Hospital and their affiliated 10 hospitals. Details of the study design and its implementation have been reported previously (15, 16).

In the current study, the time-weighted average (TWA) TyG index and CHG_TWA were assessed by data from the 2006 survey to 2010 survey, and their associations with CVD and its subtypes were assessed. This study initially included 101,510 participants who participated in the 2006 survey of the Kailuan Study. Of these, 5,591 were excluded because of missing data required to define CKM syndrome, including fast blood glucose (FBG), total cholesterol (TC), triglyceride (TG), high-density lipoprotein cholesterol (HDL-C), low-density lipoprotein cholesterol (LDL-C), waist circumference (WC), body mass index (BMI), systolic blood pressure (SBP), diastolic blood pressure (DBP), smoking status, proteinuria, and estimated glomerular filtration rate (eGFR), 3,938 with CKM syndrome stage 4 were also excluded. Next, we excluded participants who did not attend the 2008 or 2010 surveys, had missing data on SBP, DBP, FBG, TC, TG, LDL-C, HDL-C, WC, BMI, eGFR, proteinuria, and smoking status at either 2008 survey or 2010 survey, or had CKM syndrome stage 4 at the 2008 or 2010 survey. Finally, 46,827 participants who completed 2006, 2008, and 2010 surveys, had complete information required to define CKM syndrome, and were classified as CKM syndrome stages 0–3 were included in the final analysis (**Figure 1**).

**Figure 1:**
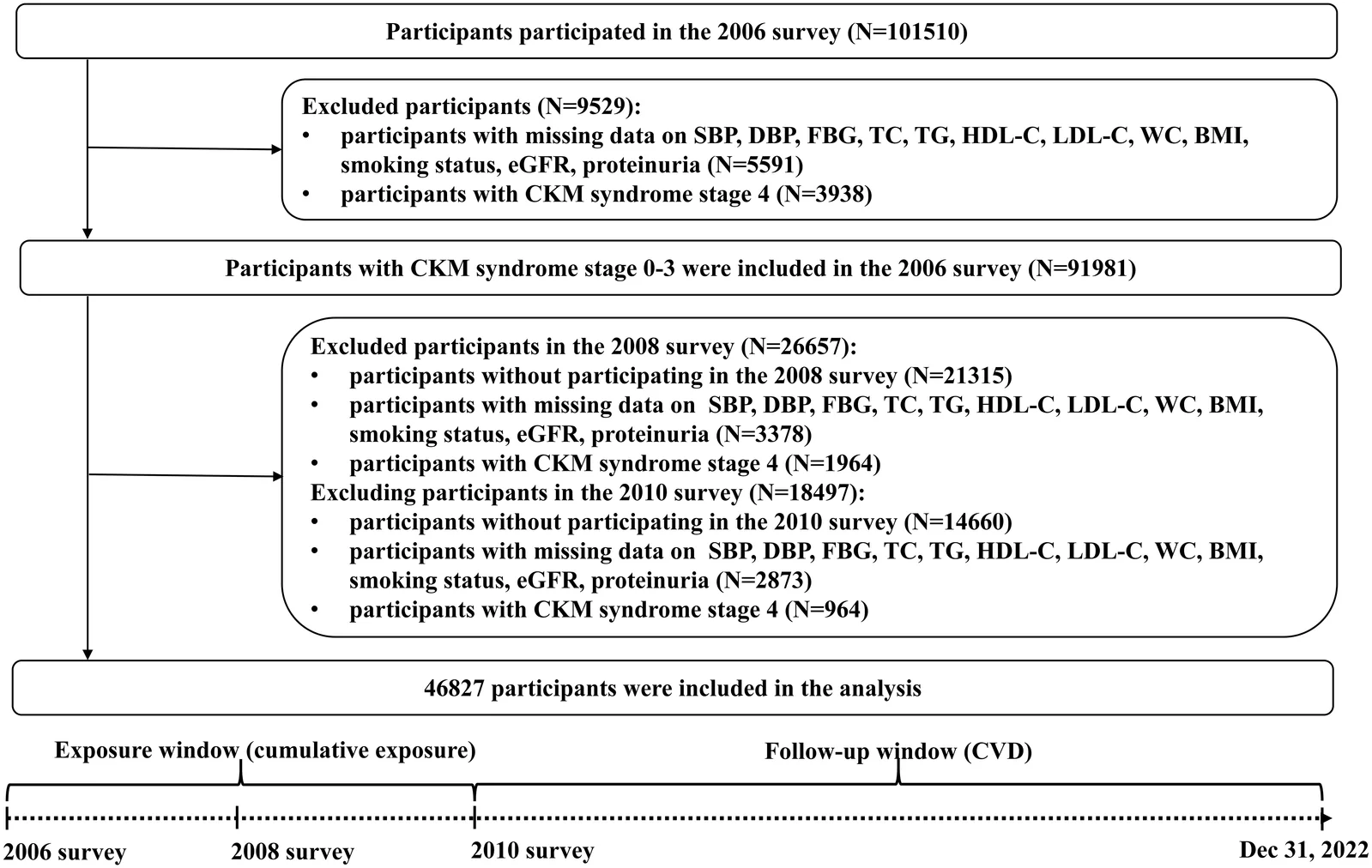
Flowchart of the participants in the current analysis. SBP, systolic blood pressure; DBP, diastolic blood pressure; FBG, fast blood glucose; TC, total cholesterol; TG, triglyceride; HDL-C, high-density lipoprotein cholesterol; LDL-C, low-density lipoprotein cholesterol; WC, waist circumference; BMI, body mass index; eGFR, estimated glomerular filtration rate; CVD, cardiovascular disease; CKM, cardiovascular-kidney-metabolic.

The protocol complied with the Declaration of Helsinki and received approval from the Ethics Committee of Kailuan General Hospital; each participant provided written informed consent.

### Data collection

Information on demographic characteristics, medical history (histories of CVD and cancer), medications use (antihypertensive, hypoglycemic, and lipid-lowering medications), and lifestyles (smoking status, alcohol consumption, and physical exercise) was collected by specialists using face-to-face questionnaires, as described elsewhere (16). Educational status was divided into high school or above, and below high school. Individuals who consumed alcohol daily for the past year were classified as current drinkers, while those who smoked at least one cigarette per day for the past year were considered current smokers. Active physical activity was defined as engaging in exercise at least four times per week, with each session lasting at least 20 minutes. Weight, height, WC, and blood pressure were measured by trained medical staff according to standardized protocols (16). BMI was derived using the standard formula: weight in kilograms divided by height squared (m²). Participants were categorized as having BMI <24 kg/m^2^ and ≥24 kg/m^2^.

On the morning of the health examination, trained medical staff collect blood samples from participants who had fasted for at least 8 hours. Biochemical indices, including creatinine, TC, TG, LDL-C, HDL-C, FBG, and high-sensitivity C-reactive protein (hs-CRP) were analyzed using an automated analyzer (Hitachi 747; Hitachi, Tokyo, Japan) (17). All participants were classified into two groups according hs-CRP levels (<3 mg/L and ≥3 mg/L). The eGFR was estimated using the Chronic Kidney Disease Epidemiology Collaboration formula (18). On the health examination day, a 5-mL clean-catch midstream morning urine sample was collected from each participant and analyzed using a urine analyzer (N-600; Changchun Dirui Medical Technology Co., Ltd.) in strict accordance with the manufacturer’s instructions. Semiquantitative proteinuria was classified as five distinct grades, ranging from negative (<15 mg/dL) and trace (15–29 mg/dL) to progressive levels of 1+ to 3+ (30–300 mg/dL, 300–1000 mg/dL, and ≥1000 mg/dL).

### Assessment of TyG_TWA and CHG_TWA and definition of high TyG index and high CHG index

TyG index and CHG index were calculated as follows (11, 19):


TyG index=Ln [TG (mg/dL)×FBG (mg/dL)/2]

CHG index=Ln [TC (mg/dL)×FBG (mg/dL)/2×HDL (mg/dL)].

Cumulative exposure was assessed using the TWA, calculated as the sum of the products of the mean value between two consecutive surveys and the corresponding time interval, divided by the total exposure duration (20), as follows:


$$\text{TyG}\_\text{TWA}=[\left(\text{Ty}{\text{G}}_{2006}+\text{Ty}{\text{G}}_{2008}\right)/2\times \text{tim}{\text{e}}_{2006-2008}+\left(\text{Ty}{\text{G}}_{2008}+\text{Ty}{\text{G}}_{2010}\right)/2\times \text{tim}{\text{e}}_{2008-2010}]/\text{tim}{\text{e}}_{2006-2010}$$

$$\text{CHG}\_\text{TWA}=[\left(\text{CH}{\text{G}}_{2006}+\text{CH}{\text{G}}_{2008}\right)/2\times \text{tim}{\text{e}}_{2006-2008}+\left(\text{CH}{\text{G}}_{2008}+\text{CH}{\text{G}}_{2010}\right)/2\times \text{tim}{\text{e}}_{2008-2010}]/\text{tim}{\text{e}}_{2006-2010},$$

where TyG_2006_, CHG_2006_, TyG_2008_, CHG_2008_, TyG_2010_, CHG_2010_ indicated TyG index and CHG index at the first (2006) survey, second (2008) survey, and third (2010) survey; time_2006–2008_ and time_2008–2010_ indicated corresponding intervals between consecutive surveys; time_2006–2010_ indicated the total exposure duration, calculated as the interval between the 2006 survey and the 2010 survey.

Participants were categorized into four groups according to quartiles (Q) of TyG_TWA or CHG_TWA:

TyG_TWA: Q1 (<8.26, reference), Q2 (8.26-<8.61), Q3 (8.61-<9.01), and Q4 (≥9.01);
CHG_TWA: Q1 (<4.89, reference), Q2 (4.89-<5.06), Q3 (5.06-<5.23), and Q4 (≥5.23).

The cut-off values for defining high TyG index and high CHG index are provided in the statistical analysis section. The exposure duration of high TyG index or CHG index was defined according to the number of surveys at which high TyG index/CHG index was observed during the exposure period. In the Kailuan Study, surveys were conducted approximately every 2 years. Therefore, participants were categorized as having 0 years (without high TyG index/CHG index at any survey, reference), 2 years (high TyG index/CHG index at one survey), 4 years (high TyG index/CHG index at two surveys), and 6 years (high TyG index/CHG index at three surveys) of exposure duration.

### Assessment of CVD and definitions

Participants were followed from the 2010 survey until the first occurrence of incident CVD, all-cause mortality, or 31 December 2022, whichever came first. In addition to the biennial surveys, cardiovascular events, including stroke (I60, I61, and I63), myocardial infarction (MI, I21), heart failure (HF, I50), and atrial fibrillation (AF, I48), were ascertained annually from the Municipal Social Insurance Institution and the social security system of the Kailuan Group. Cardiovascular events were diagnosed and coded by trained physicians according to the International Classification of Diseases, 10th Revision (ICD-10) (21). The definitions of cardiovascular events were further supported by previously established criteria for stroke, MI, HF, and AF (22–25). All-cause mortality was assessed annually by professional physicians based on death certificate from the provincial population statistics office (26).

Hypertension was defined as a self-reported history of hypertension, use of antihypertensive medication, SBP≥140 mmHg and/or DBP≥90 mmHg (27). Diabetes mellitus was defined as a history of diabetes mellitus, current use of hypoglycemic medication, or FBG≥7 mmol/L (28). For multivariable analyses, dyslipidemia was defined as TC≥6.2 mmol/L, TG≥2.3 mmol/L, LDL-C≥4.1 mmol/L, HDL-C<1.04 mmol/L, or the use of lipid-lowering medication, and was treated as a covariate in the multivariable models. For CKM syndrome classification, dyslipidemia was defined as the use of lipid-lowering medication, or the presence of any of the following criteria: TC≥5.2 mmol/L, TG≥1.7 mmol/L, LDL-C≥3.4 mmol/L, or HDL-C<1.00 mmol/L (29). CKD risk categories (low, moderate, high, and very high risk) were defined according to the Kidney Disease Improving Global Outcomes 2012 recommendations (30). Metabolic syndrome was defined as the presence of ≥3 of the following criteria (1): (1) WC≥80 cm for females or ≥90 cm for males; (2) SBP/DBP≥130/80 mmHg or the use of antihypertensive medication; (3) FBG≥5.6 mmol/L or the use of hypoglycemic medication; (4) HDL-C<1.04 mmol/L for males and <1.29 mmol/L for females; (5) TG≥ 1.7 mmol/L or the use of lipid-lowering medication.

### Definition of CKM syndrome

According to the scientific statement from the AHA in 2023, CKM syndrome was classified into five stages (1): Stage 0 was defined as no CKM risk factors and characterized by normal BMI and WC, normoglycemia, normotension, a normal lipid profile, and no evidence of CKD or subclinical or clinical CVD. Stage 1 was defined as having overweight/obesity (BMI≥23 kg/m^2^), abdominal obesity (WC≥80 cm for females and WC≥90 cm for males), or dysfunctional adipose tissue (FBG≥5.6-6.9 mmol/L), without the other metabolic risk factors or CKD. Stage 2 was defined as the presence of metabolic risk factors (TG≥1.5 mmol/L, dyslipidemia, hypertension, metabolic syndrome, diabetes mellitus), or moderate-to-high risk CKD. Stage 3 was defined as subclinical CVD among individuals with excess/dysfunctional adiposity, other metabolic risk factors, or CKD. This study used the risk equivalents to define stage 3 due to the absence of related data. CKM stage 3 was defined as very high-risk CKD or high predicted 10-year CVD risk calculated by the Prediction for Atherosclerotic Cardiovascular Disease Risk in China (31). Stage 4 was defined as clinical CVD (stroke, MI, HF, AF).

### Statistical analyses

The CVD-related optimal thresholds for TyG_TWA and CHG_TWA were identified using a survival-time method to calculate maximally selected rank statistics, implement with the maxstat package and relevant packages as previously described (32).

Continuous variables were reported as mean ± standard deviation (SD) or median (interquartile range), as appropriate, and were compared using independent-samples t test or Mann-Whitney U test. Categorical variables were expressed as number (percentage) and compared using Chi-square test. Missing covariate data (<5%) were handled using the chain equations to impute.

The cumulative incidence of CVD was assessed using the Kaplan-Meier method, and differences were assessed with the Log-rank test. Incidence rates of CVD and subtypes were calculated and expressed per 1000 person-years. Multivariable Cox proportional hazards regression models (hazard ratio, HR and 95% confidence interval, 95% CI) were used to assess the associations of TyG_TWA, CHG_TWA, exposure duration of high TyG index, and exposure duration of high CHG index with the risk of CVD among participants with CKM syndrome stages 0-3. The associations between per SD increment in TyG_TWA/CHG_TWA and CVD risk were assessed. Model 1 adjusted for age and gender. Model 2 adjusted for covariates in model 1 plus BMI categories, drinking status, smoking status, physical exercise, and educational status. Model 3 adjusted for covariates in model 2 plus hs-CRP categories, CKD, and hypertension. Model 4 adjusted for covariates in model 3 plus diabetes mellitus and dyslipidemia. We treated categorized variable as a continuous variable to test P for trend. We further assessed the associations of per SD increment in TyG_TWA and CHG_TWA with the risk of CVD among participants across different CKM syndrome stages. The dose-response associations between continuous TyG_TWA/CHG_TWA and the risk of CVD were assessed using restricted cubic splines (RCS) analysis with three knots (25th, 50th, 75th percentiles). Receiver operating characteristic (ROC) curves were constructed to assess the predictive performances of TyG_TWA and CHG_TWA for CVD by comparing the area under the curve (AUC). The optimal cut-off values were calculated using the Youden index.

To assess potential effect modification, we conducted subgroup analyses for the association of per SD increment in TyG_TWA or CHG_TWA with the risk of CVD among participants with CKM syndrome stages 0–3 by age (<60 years and ≥60 years), sex (males and females), BMI (<24 kg/m^2^ and ≥24 kg/m^2^), hs-CRP (<3 mg/L and ≥3 mg/L), hypertension (yes and no), diabetes mellitus (yes and no), and dyslipidemia (yes and no). Multiplicative interactions were assessed via Z test, we also compared the ratio of HR (95% CI) between two groups. To test the robustness of results, we conducted several sensitivity analyses: (1) excluding participants with incident CVD within 2 years; (2) excluding participants with antihypertensive medications, hypoglycemic medications, or lipid-lowering medications; (3) excluding participants with a history of cancer; (4) we used competing risk model, considering death as a competing risk. In addition, among participants with CKM syndrome stages 0-2, the associations of cumulative exposure to TyG index and CHG index with CVD risk were reassessed using the same multivariable Cox regression models as those used in the primary analyses.

All statistical analyses were conducted using SAS version 9.4 (SAS Institute, Cary, NC) and R version 4.4.2, two-sided P value< 0.05 were considered statistically significant.

## Results

### Baseline characteristics

Among 46,827 participants, the mean age was (52.71 ± 11.81) years, 76.80% were males, 9.91% developed CVDs during follow-up. Compared with the participants who remained free of CVD, those who developed CVD were older and less-educated, had BMI, FBG, TG, TC, hs-CRP, TyG_TWA, and CHG_TWA, had a lower HDL-C, had higher prevalence of hypertension, diabetes mellitus, dyslipidemia, CKM stage 3, and higher usage of antihypertensive medications, hypoglycemic medications, lipid-lowering medications. The comparisons of all participants according to CVD incident are presented in **Table 1**.

**Table 1:** Baseline characteristics of participants according to CVD incidence.

Characteristics	Overall	CVD incidence		P value
		No	Yes	
Number of participants	46827	42188	4639	–
Age, years	52.71 ± 11.81	52.02 ± 11.74	59.01 ± 10.48	<0.05
Male, n (%)	35963 (76.80)	32031 (75.92)	3932 (84.76)	<0.05
BMI, kg/m^2^	25.32 ± 3.42	25.25 ± 3.41	25.97 ± 3.44	<0.05
FBG, mmol/L	5.63 ± 1.50	5.58 ± 1.42	6.06 ± 1.99	<0.05
HDL-C, mmol/L	1.55 ± 0.47	1.55 ± 0.48	1.51 ± 0.45	<0.05
TG, mmol/L	1.29 (0.91, 1.91)	1.28 (0.91, 1.90)	1.37 (0.98, 2.05)	<0.05
TC, mmol/L	4.98 ± 0.99	4.96 ± 0.99	5.12 ± 1.03	<0.05
Hs-CRP, mg/L	1.06 (0.50, 2.54)	1.01 (0.49, 2.49)	1.30 (0.60, 3.13)	<0.05
TyG_TWA	8.67 ± 0.59	8.65 ± 0.59	8.81 ± 0.61	<0.05
CHG_TWA	5.08 ± 0.30	5.07 ± 0.30	5.18 ± 0.34	<0.05
Current smokers, n (%)	15881 (33.91)	14246 (33.77)	1635 (35.24)	<0.05
Current drinkers, n (%)	16448 (35.13)	14909 (35.34)	1539 (33.18)	<0.05
Active physical activity, n (%)	6554 (14.00)	5824 (13.80)	730 (15.74)	<0.05
High school or above, n (%)	10717 (22.89)	10049 (23.82)	668 (14.40)	<0.05
CKD, n (%)	3044 (6.50)	2582 (6.12)	462 (9.96)	<0.05
Diabetes mellitus, n (%)	5104 (10.90)	4136 (9.80)	968 (20.87)	<0.05
Hypertension, n (%)	22073 (47.14)	18905 (44.81)	3168 (68.29)	<0.05
Dyslipidemia, n (%)	14279 (30.49)	12630 (29.94)	1649 (35.55)	<0.05
Antihypertensive medications, n (%)	7520 (16.06)	6296 (14.92)	1224 (26.38)	<0.05
Hypoglycemic medications, n (%)	1789 (3.82)	1403 (3.33)	386 (8.32)	<0.05
Lipid-lowering medications, n (%)	400 (0.85)	343 (0.81)	57 (1.23)	<0.05
CKM syndrome stage at 2006/2007 survey, n (%)				<0.05
Stage 0	3567 (7.62)	3435 (8.14)	132 (2.85)	
Stage 1	6731 (14.37)	6389 (15.14)	342 (7.37)	
Stage 2	29735 (63.50)	26992 (63.98)	2743 (59.13)	
Stage 3	6794 (14.51)	5372 (12.73)	1422 (30.65)	
CKM syndrome stage at 2010/2011 survey, n (%)				<0.05
Stage 0	2960 (6.32)	2879 (6.82)	81 (1.75)	
Stage 1	6097 (13.02)	5817 (13.79)	280 (6.04)	
Stage 2	26946 (57.54)	24788 (58.76)	2158 (46.52)	
Stage 3	10824 (23.11)	8704 (20.63)	2120 (45.70)	

CVD, cardiovascular disease; BMI, body mass index; FBG, fast blood glucose; HDL-C, high-density lipoprotein cholesterol; TC, total cholesterol; TG, triglyceride; hs-CRP, high-sensitive C-reactive protein; TyG, triglyceride-glucose; CHG, cholesterol, high-density lipoprotein, and glucose; TWA, time-weighted average; CKD, chronic kidney disease; CKM, cardiovascular-kidney-metabolic.

### Association of TyG_TWA, CHG_TWA, and exposure duration of high TyG index/CHG index with the risk of CVD

Over a mean follow-up of 11.08 (SD 2.49) years, 4,639 participants developed CVD, of these, 3011 developed stroke, 702 developed MI, 907 developed HF, 435 developed AF. The incidence rates of CVD increased significantly with the increase of the Q of TyG_TWA/CHG_TWA and exposure duration of high TyG index/CHG index. Similar results also were shown in Kaplan-Meier curves (Log-rank test, P<0.05; **Figure 2**). In model 1, participants in the Q4 group of TyG_TWA had a higher risk of CVD compared with those in Q1 group (HR, 1.95; 95% CI, 1.79-2.12); the corresponding HRs were 1.82 (95% CI, 1.67-1.98) in model 2, 1.64 (95% CI, 1.51-1.79) in model 3, and 1.36 (95% CI, 1.23-1.50) in model 4. With per SD increment in TyG_TWA, HRs for CVD were increased by 30% (95% CI, 1.27-1.34) in model 1, 27% (95% CI, 1.24-1.31) in model 2, 23% (95% CI, 1.19-1.26) in model 3, and 14% (95% CI, 1.10-1.18) in model 4. Similarly, in model 4, compared to participants in Q1 group of CHG_TWA, the HRs of CVD were 1.04 (95% CI, 0.95-1.14) for those in Q2 group, 1.13 (95% CI, 1.04-1.24) for those in Q3 group, and 1.23 (95% CI, 1.12-1.36) for those in Q4 group. With per SD increment in CHG_TWA, HRs for CVD were increased by 13% (95% CI, 1.09-1.17) in model 4, which was similar to the results in model 1, model 2 and model 3. Above results can be found in **Table 2**. In addition, there were linear associations between TyG_TWA and risk of CVD in four models, the associations of CHG_TWA with the risk of CVD were linear in model 1, model 2, and model 3, whereas this association was non-linear in model 4 (**Figure 3**).

**Figure 2:**
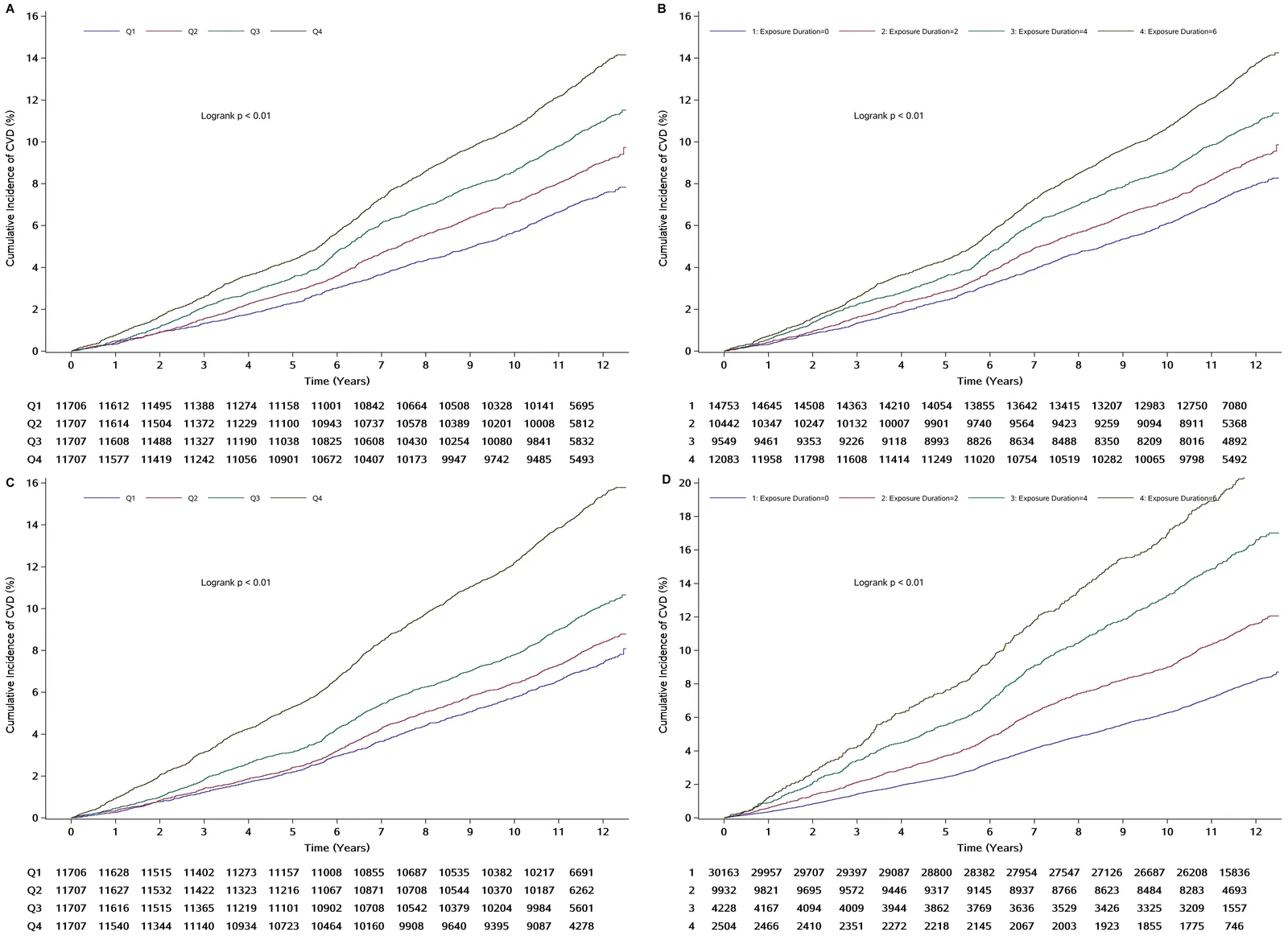
Kaplan-Meier curves of CVD by quartiles of TyG_TWA/CHG_TWA and exposure duration of high TyG index/CHG index. **(A)** TyG_TWA and CVD; **(B)** Exposure duration of high TyG index and CVD; **(C)** CHG_TWA and CVD; **(D)** Exposure duration of high CHG index and CVD. Q, quartile; CVD, cardiovascular disease; TyG, triglyceride-glucose; CHG, cholesterol, high-density lipoprotein, and glucose; TWA, time-weighted average.

**Table 2:** Association of TyG_TWA, CHG_TWA, exposure duration of high TyG index, and exposure duration of high CHG index with the risk of CVD among participants with CKM syndrome stages 0-3.

Exposure	Case/Total	Incidence ratio	Model 1	Model 2	Model 3	Model 4
TyG_TWA						
Q1	843/11706	6.41 (6.00-6.86)	Reference	Reference	Reference	Reference
Q2	1019/11707	7.80 (7.33-8.29)	1.19 (1.09-1.31)	1.16 (1.06-1.27)	1.12 (1.03-1.23)	1.10 (1.01-1.21)
Q3	1243/11707	9.59 (9.07-10.14)	1.49 (1.36-1.62)	1.41 (1.29-1.54)	1.33 (1.21-1.45)	1.24 (1.14-1.36)
Q4	1534/11707	12.06 (11.47-12.68)	1.95 (1.79-2.12)	1.82 (1.67-1.98)	1.64 (1.51-1.79)	1.36 (1.23-1.50)
P for trend	–	–	<0.05	<0.05	<0.05	<0.05
Per SD increment	4639/46827	8.94 (8.69-9.20)	1.30 (1.27-1.34)	1.27 (1.24-1.31)	1.23 (1.19-1.26)	1.14 (1.10-1.18)
Exposure duration of high TyG index						
0 years	1126/14753	6.81 (6.42-7.22)	Reference	Reference	Reference	Reference
2 years	925/10442	7.93 (7.44-8.46)	1.21 (1.11-1.32)	1.18 (1.08-1.28)	1.13 (1.03-1.23)	1.09 (1.00-1.19)
4 years	1007/9549	9.53 (8.96-10.14)	1.47 (1.35-1.60)	1.39 (1.28-1.52)	1.31 (1.20-1.42)	1.21 (1.10-1.32)
6 years	1581/12083	12.04 (11.46-12.65)	1.82 (1.68-1.96)	1.70 (1.57-1.84)	1.54 (1.42-1.67)	1.28 (1.16-1.40)
P for trend	**-**	**-**	<0.05	<0.05	<0.05	<0.05
CHG_TWA						
Q1	845/11706	6.39 (5.97-6.83)	Reference	Reference	Reference	Reference
Q2	952/11707	7.20 (6.76-7.67)	1.11 (1.01-1.21)	1.07 (0.98-1.18)	1.06 (0.96-1.16)	1.04 (0.95-1.14)
Q3	1148/11707	8.81 (8.31-9.33)	1.30 (1.19-1.42)	1.23 (1.12-1.35)	1.18 (1.08-1.30)	1.13 (1.04-1.24)
Q4	1694/11707	13.65 (13.02-14.32)	1.77 (1.63-1.93)	1.65 (1.51-1.79)	1.54 (1.41-1.67)	1.23 (1.12-1.36)
P for trend	–	–	<0.05	<0.05	<0.05	<0.05
Per SD increment	4639/46827	8.94 (8.69-9.20)	1.29 (1.26-1.32)	1.26 (1.23-1.30)	1.23 (1.20-1.26)	1.13 (1.09-1.17)
Exposure duration of high CHG index						
0 years	2389/30163	7.03 (6.75-7.32)	Reference	Reference	Reference	Reference
2 years	1108/9932	10.15 (9.57-10.76)	1.39 (1.29-1.49)	1.34 (1.25-1.44)	1.30 (1.21-1.40)	1.22 (1.13-1.31)
4 years	655/4228	14.73 (13.65-15.91)	1.74 (1.59-1.89)	1.65 (1.51-1.80)	1.55 (1.42-1.69)	1.30 (1.18-1.44)
6 years	487/2504	19.15 (17.52-20.93)	2.20 (2.00-2.43)	2.08 (1.88-2.29)	1.92 (1.74-2.12)	1.41 (1.25-1.59)
P for trend	–	–	<0.05	<0.05	<0.05	<0.05

CVD, cardiovascular disease; BMI, body mass index; FBG, fast blood glucose; HDL-C, high-density lipoprotein cholesterol; TC, total cholesterol; TG, triglyceride; hs-CRP, high-sensitive C-reactive protein; eGFR, estimated glomerular filtration rate; TyG, triglyceride-glucose; CHG, cholesterol, high-density lipoprotein, and glucose; TWA, time-weighted average; CKM, cardiovascular-kidney-metabolic; CKD, chronic kidney disease. Incidence ratio is per 1000 person-years (95% CI).

Model 1 adjusted for age and gender.

Model 2 adjusted for covariates in model 1 plus BMI categories, drinking status, smoking status, physical exercise, and educational status.

Model 3 adjusted for covariates in model 2 plus hs-CRP categories, CKD, and hypertension.

Model 4 adjusted for covariates in model 3 plus diabetes mellitus and dyslipidemia.

**Figure 3:**
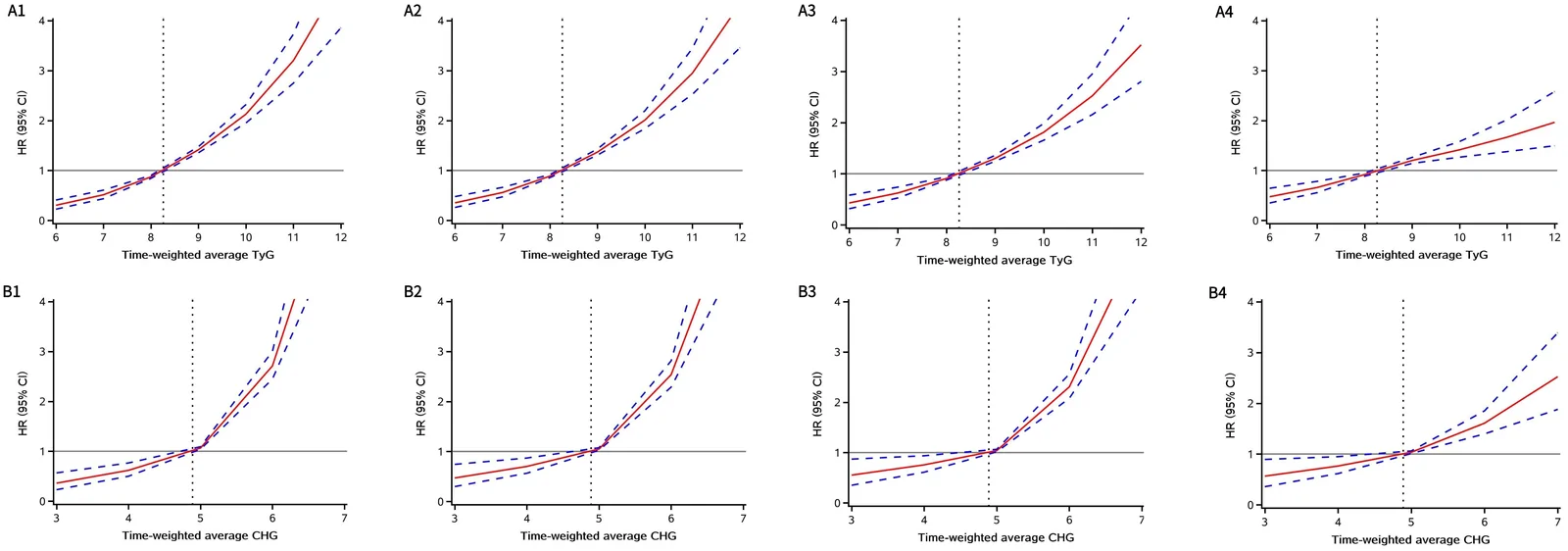
HR and 95%CI for the association of TyG_TWA and CHG_TWA with the risk of CVD by using restricted cubic spline regression with 3 knots with placed at the 25th, 50th, and 75th percentiles. **(A1–A4)** TyG_TWA and the risk of CVD in model 1, model 2, model 3, and model 4; **(B1–B4)** CHG_TWA and the risk of CVD in model 1, model 2, model 3, and model 4. The red lines indicate HR, the blue lines indicate 95% CI. CVD, cardiovascular disease; TyG, triglyceride-glucose; CHG, cholesterol, high-density lipoprotein, and glucose; TWA, time-weighted average; HR, hazard ratio; CI, confidence interval; BMI, body mass index; hs-CRP, high-sensitive C-reactive protein; CKD, chronic kidney disease. Model 1 adjusted for age and gender; Model 2 adjusted for covariates in model 1 plus BMI categories, drinking status, smoking status, physical exercise, and educational status; Model 3 adjusted for covariates in model 2 plus hs-CRP categories, CKD, and hypertension; Model 4 adjusted for covariates in model 3 plus diabetes mellitus and dyslipidemia.

The optimal cut-off values of TyG_TWA and CHG_TWA associated with CVD incident were 8.65 and 5.36 (**Supplementary Figure S1**). The participants who had a longer exposure duration of high TyG index and high CHG index had a higher risk of CVD incident. In model 3, compared with participants without exposure of high TyG index, the HR of CVD was increased by 54% (95% CI, 1.42-1.67) for those with 6 years exposure of high TyG index; compared with participants without exposure of high CHG index, the HR of CVD was increased by 92% (95% CI, 1.74-2.12) for those with 6 years exposure of high CHG index. After adjusting for all potential covariates, compared with participants without exposure of high TyG index, the HRs of CVD were increased by 9% (95% CI, 1.00-1.19) for those with 2 years exposure of high TyG index, 21% (95% CI, 1.10-1.32) for those with 4 years exposure of high TyG index, 28% (95% CI, 1.16-1.40) for those with 6 years exposure of high TyG index. Compared with participants without exposure of high CHG index, the HRs of CVD were increased by 22% (95% CI, 1.13-1.31) for those with 2 years exposure of high CHG index, 30% (95% CI, 1.18-1.44) for those with 4 years exposure of high CHG index, 41% (95% CI, 1.25-1.59) for those with 6 years exposure of high CHG index.

We further observed the associations of cumulative TyG index/CHG index with CVD subtypes (**Supplementary Tables S1**–**S4**). The associations with the stroke and MI were still consistent after adjusted for all covariates. However, associations of per SD increment in TyG_TWA and CHG_TWA were also observed for HF. The results of RCS analysis showed that there were linear associations of TyG_TWA/CHG_TWA with risk of stroke in model 4, the associations of TyG_TWA/CHG_TWA with the risk of MI were non-linear in model 4 (**Supplementary Figures S2**, **S3**). There was a linear association between the CHG_TWA and the risk of HF, the associations between TyG_TWA/CHG_TWA and the risk of AF or TyG_TWA and HF were not significant in RCS analysis.

### Comparison of predictive performances of TyG_TWA and CHG_TWA for CVD risk

The result of ROC curve analysis showed that CHG_TWA had a modestly higher discriminatory ability for CVD risk (**Figure 4**). The AUC was 0.592 for CHG_TWA and 0.576 for TyG_TWA (**Supplementary Table S7**). The Youden index of CHG_TWA (Youdex index, 0.139) for CVD was higher than that of TyG_TWA (Youdex index, 0.115). And the P for comparison was significant.

**Figure 4:**
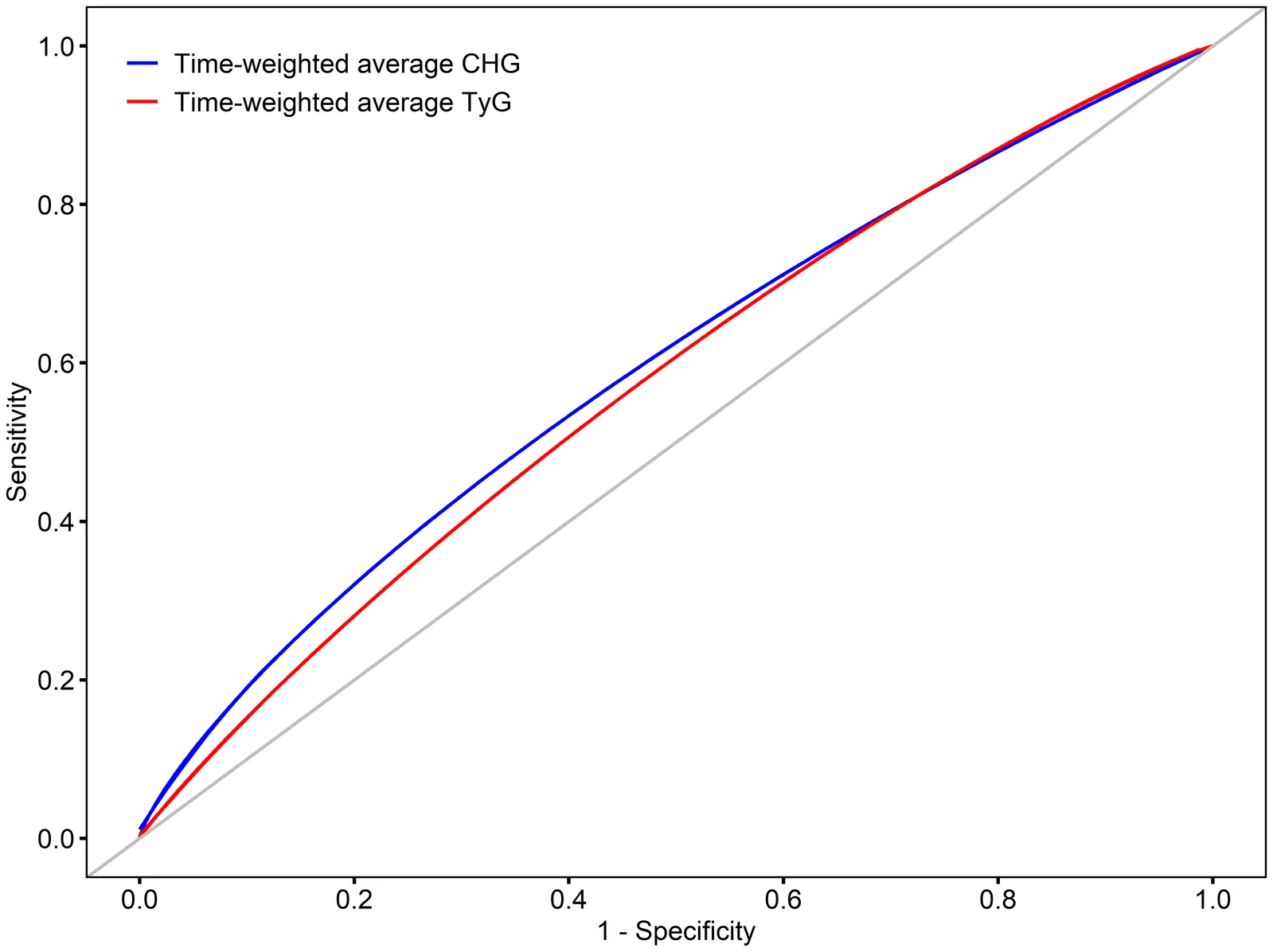
Comparison of predictive performances for CVD between TyG_TWA and CHG_TWA. The AUC for TyG_TWA is 0.576, the AUC for CHG_TWA is 0.592, and the P for comparison <0.001. The red lines indicate TyG_TWA, the blue lines indicate CHG_TWA. TyG, triglyceride-glucose; CHG, cholesterol, high-density lipoprotein, and glucose; TWA, time-weighted average; CVD, cardiovascular disease.

### Subgroup and sensitivity analyses

In the analysis across CKM syndrome stages, the associations of per SD increment in TyG_TWA or CHG_TWA with the risk of CVD were consistent to the main results whether the CKM syndrome stage at 2006 survey or 2010 survey (**Supplementary Tables S5**, **S6**). And we found this association decreased with increasing CKM syndrome stages. The multiplicative interactions were found between age and TyG_TWA/CHG_TWA on the risk of CVD (**Table 3**). There were interactions between TyG_TWA and hypertension and dyslipidemia. The HRs of CVD for per SD increment in TyG_TWA were 1.15 (95% CI, 1.10-1.20) in participants with an age<60 years versus 1.05 (95% CI, 0.99-1.11) in participants with an age≥60 years (<60 years-to-≥60 years ratio of HR: 1.10 [95% CI, 1.02-1.18]), the HRs of CVD were 1.23 (95% CI, 1.15-1.31) in participants without hypertension versus 1.09 (95% CI, 1.05-1.14) in participants with hypertension (no-to-yes ratio of HR: 1.13 [95% CI, 1.04-1.22]), the HRs of CVD were 1.20 (95% CI, 1.15-1.26) in participants without dyslipidemia versus 1.07 (95% CI, 1.02-1.13) in participants with dyslipidemia (no-to-yes ratio of HR: 1.12 [95% CI, 1.05-1.20]). The HRs of CVD for per SD increment in CHG_TWA were 1.19 (95% CI, 1.14-1.25) in participants with an age<60 years versus 1.08 (95% CI, 1.03-1.14) in participants with an age≥60 years (<60 years-to-≥60 years ratio of HR: 1.10 [95% CI, 1.03-1.18]). The result of sensitivity analysis restricted to participants with CKM syndrome stages 0–2 remained consistent with the primary analyses (**Supplementary Table S11**). Other results of sensitivity analyses were similar to main findings (**Supplementary Tables S8**–**S10**).

**Table 3:** Subgroup analyses for the association of per SD increment in TyG_TWA or CHG_TWA with the risk of CVD among participants with CKM syndrome stages 0-3.

Subgroup categories	TyG_TWA			CHG_TWA		
	HR (95% CI)	Group 1 to group 2 ratio of HR (95%CI)	P for interaction	HR (95% CI)	Group 1 to group 2 ratio of HR (95%CI)	P for interaction
Age, years						
<60	1.15 (1.10-1.20)	1.10 (1.02-1.18)	<0.05	1.19 (1.14-1.25)	1.10 (1.03-1.18)	<0.05
≥60	1.05 (0.99-1.11)			1.08 (1.03-1.14)		
Sex						
Females	1.20 (1.09-1.32)	1.07 (0.97-1.19)	0.19	1.20 (1.10-1.30)	1.08 (0.99-1.19)	0.10
Males	1.12 (1.08-1.17)			1.11 (1.07-1.16)		
BMI, kg/m^2^						
<24	1.17 (1.10-1.25)	1.04 (0.97-1.13)	0.26	1.18 (1.11-1.25)	1.06 (0.99-1.14)	0.10
≥24	1.12 (1.08-1.17)			1.11 (1.06-1.15)		
hs-CRP, mg/L						
<3	1.14 (1.10-1.19)	1.02 (0.94-1.10)	0.65	1.13 (1.08-1.17)	1.00 (0.93-1.08)	1.00
≥3	1.12 (1.05-1.20)			1.13 (1.06-1.21)		
Hypertension						
No	1.23 (1.15-1.31)	1.13 (1.04-1.22)	<0.05	1.14 (1.07-1.21)	1.02 (0.95-1.10)	0.64
Yes	1.09 (1.05-1.14)			1.12 (1.08-1.17)		
Diabetes mellitus						
No	1.13 (1.09-1.18)	0.99 (0.92-1.07)	0.82	1.12 (1.07-1.17)	0.98 (0.91-1.06)	0.63
Yes	1.14 (1.06-1.21)			1.14 (1.08-1.21)		
Dyslipidemia						
No	1.20 (1.15-1.26)	1.12 (1.05-1.20)	<0.05	1.13 (1.08-1.18)	1.01 (0.94-1.08)	0.80
Yes	1.07 (1.02-1.13)			1.12 (1.06-1.18)		

CVD, cardiovascular disease; BMI, body mass index; hs-CRP, high-sensitive C-reactive protein; TyG, triglyceride-glucose; CHG, cholesterol, high-density lipoprotein, and glucose; TWA, time-weighted average; CKD, chronic kidney disease; CKM, cardiovascular-kidney-metabolic. Models adjusted for age, gender, BMI categories, drinking status, smoking status, physical exercise, and educational status, hs-CRP categories, CKD, hypertension, diabetes mellitus, and dyslipidemia.

Group 1 includes participants who are age<60 years, females, BMI<24kg/m^2^, without hypertension, without diabetes mellitus, without dyslipidemia, or hs-CRP<3 mg/L; Group 2 includes participants who are age≥60 years, males, BMI≥24kg/m^2^, hypertension, diabetes mellitus, dyslipidemia, or hs-CRP≥3 mg/L.

## Discussion

In this study, we found that higher CHG_TWA and TyG_TWA were significantly associated with the risk of CVD among individuals with CKM syndrome stages 0-3. Notably, CHG_TWA demonstrated a modestly higher discriminatory ability for CVD risk compared with TyG_TWA. The risk of CVD increased with exposure duration of the high CHG index and TyG index. These findings suggest that longitudinal assessment of CHG index and TyG index may provide additional information for CVD risk stratification and highlight the potential importance of early identification and management of metabolic abnormalities among individuals with CKM syndrome stages 0-3.

Although previous studies have established the associations of both baseline TyG index and cumulative TyG index with the risk of CVD (33–35), emerging evidence also indicated that increased TyG index at baseline was associated with the risk of CVD among populations with CKM syndrome stages 0–3 (36, 37). Nevertheless, our findings provided compelling evidence that TyG_TWA had an independent discriminatory ability for CVD among populations with CKM syndrome stages 0-3. We found that higher TyG_TWA was closely associated with an increased risk of CVD, and this risk further increased with exposure duration of high TyG index. Currently, the majority of the studies mainly focused on the association between baseline TyG index and CVD risk among populations with CKM syndrome stages 0-3, with findings consistently supporting a positive association (36, 38). Cumulative exposure offers a comprehensive assessment of long-term fluctuation and intensity, however, the associations of TyG_TWA and exposure duration of high TyG index with the risk of CVD in populations CKM syndrome stages 0–3 still needs further study (39). Our study provides robust evidence for the association between longitudinal changes in TyG index and CVD risk among CKM syndrome stages 0-3.

IR refers to the reduced capacity to uptake glucose in target tissues such as skeletal muscles, adipose tissue, and liver in the face of physiological insulin concentrations, and has been widely confirmed as a critical factor of increase CVD risk (40–42). The hyperinsulinemic-euglycemic clamp test, the gold standard for assessing IR, is not applicable to population screening owing to complicated operation, high cost, and so on (43). In comparison, the homeostasis model assessment of insulin resistance (HOMA-IR), which is calculated from fasting glucose and fasting insulin levels, has long been regarded as a simple, reliable, and reproducible measure for assessing IR in both epidemiological studies and clinical practice (44). However, compared with the TyG index, which has recently emerged as a surrogate of IR, HOMA-IR presents several limitations, including high cost and limited utility in individuals receiving insulin therapy or those lacking functional β-cells (45, 46). In 2025, Mansoori et al. proposed the CHG index as a simple surrogate of IR and demonstrated its superior diagnostic performance for type 2 diabetes compared with the TyG index (11). To the best of our knowledge, the association between higher CHG index at baseline and increased CVD risk was observed in one study only, and the CHG index had a higher predictive performance compared to the TyG index (12). However, this study was also limited by a small sample size, lack of detailed CVD subtypes, and based on a single measurement of CHG index. Hence, further evidence is needed to clarify the potential role of the CHG index in CVD risk and to assess its discriminatory ability for CVD risk. This is the first large prospective cohort study examining the association between cumulative exposure to CHG index and CVD risk among populations with CKM syndrome stages 0-3, and comparing discriminatory abilities of TyG index and CHG index for CVD risk. In the present study, we integrated the TWA level and exposure duration to reflect longitudinal changes, and found elevated CHG_TWA and exposure duration of high CHG index in populations with CKM syndrome stages 0–3 were significantly associated with an increased risk of CVD and its subtypes including stroke, MI, and HF. In addition, we determined the optimal cut-off values of TyG_TWA and CHG_TWA associated with an increased risk of CVD, and we found that the CVD risk substantially increased when TyG_TWA exceeded 8.65 and CHG_TWA exceeded 5.36. Our study supports the conclusion that the CHG index has a modestly discriminatory ability for CVD risk among populations with CKM syndrome stages 0–3 compared with TyG index. Overall, as a simple, economical, straightforward, and convenient surrogate of IR and metabolic dysfunction, longitudinal assessment of changes in CHG index may provide additional insights into CVD risk stratification in epidemiological studies and clinical practice. Consequently, this index establishes a robust basis for early intervention and the development of personalized management strategies for patients with CKM syndrome. And this provides a strong foundation for early interventions and personalized management strategies in populations with CKM syndrome. Identifying optimal cut-off points associated with an increased risk of CVD will provide valuable insights into the burden of high CHG index. The components of the CHG index, including TC, FBG, and HDL-C, may serve as targets for lifestyle modifications and pharmacological interventions, thereby contributing to prevent the onset and development of disease.

Another important finding of this study is that the association between TyG_TWA and CVD risk is significant in individuals with CKM syndrome stages 0–2 but not in stage 3, whereas the effect of CHG_TWA on CVD risk gradually attenuated with increasing CKM syndrome stages. CKM syndrome is a progressive condition that encompasses numerous key factors contributing to the development of CVD and highlights the interplay between the heart, kidneys, and metabolism (1, 2, 47). Adipose tissue excess and dysfunction frequently advance to metabolic risk factors and CKD (48), subsequently facilitating the development of coronary atherosclerosis, subclinical abnormalities in myocardial structure and function, and a progression decline in renal function (35). These conditions ultimately lead to an increased risk of clinical CVD, renal failure, disability, and mortality (1). Although the discriminatory abilities of TyG index and CHG index for CVD risk in patients with target organ damage was weak, TyG index and CHG index remain the risk factors for CVD development in the early stages of CKM syndrome, and monitoring and intervening on the two indices may lead to a greater clinical benefit.

We also found that the associations of TyG_TWA and CHG_TWA with CVD risk were consistent across all subgroups. However, TyG_TWA and CHG_TWA showed stronger associations with CVD risk in younger and middle-aged populations than in older adults. Currently, several potential mechanisms have been proposed to explain how age influences the relationship between IR and CVD. First, younger and middle-aged populations generally have greater β-cell compensatory capacity and metabolic reserves, which allow for the maintenance of glucose homeostasis during early-stage IR through increased insulin secretion. However, with the accumulation of IR, compensatory mechanisms gradually become overwhelmed, resulting in earlier onset of metabolic disturbances and vascular damage. Consequently, this leads to an increased risk of CVD within a shorter time frame (49, 50). Conversely, older populations frequently display chronic vascular stiffness and endothelial dysfunction, CVD events primarily influenced by other significant risk factors. Consequently, the incremental risk associated with IR is comparatively diminished in this population (50, 51). Second, there are obvious age-related differences in the vascular plasticity. In younger and middle-aged individuals, vascular structures exhibit greater reversibility, and IR-associated adverse metabolic changes (such as dyslipidemia, inflammation, and oxidative stress) can expedite the progression of atherosclerosis; in older adults, these pathological processes are typically more advanced, and CVD events are predominantly influenced by other significant risk factors, which diminishes the relative impact of IR (52, 53). Simultaneously, as individuals age, there is a general increase in chronic low-grade inflammation and visceral adiposity. Consequently, the pro-inflammatory effects of IR tend to be relatively diminished in older adults, younger and middle-aged populations exhibit greater susceptibility to these effects (54). Finally, competing risks and survivor bias may partially explain the attenuated effects of IR in older individuals. Older populations often die from non-CVD causes and experience CVD events at earlier stages which can lead to a reduction in the observed relative risk (55). These findings suggest that younger and middle-aged stage may represent a critical window for CVD prevention, highlighting the need to strengthen screening and management of IR, and to delay the progression of atherosclerosis through lifestyle or pharmacological interventions. In older populations, management should focus on controlling IR while comprehensively addressing blood pressure, lipid levels, and other chronic conditions to reduce the overall CVD burden.

The strengths of this study are as follows. First, it is a prospective cohort study with a mean follow-up period of 11.08 years, focusing on participants with CKM syndrome stages 0–3 rather than the general population. Second, we used TWA levels to elucidate longitudinal changes in exposure variables and fully considered the optimal cutoffs associated with increased CVD risk, which may provide insights surpassing those obtained from single measurements. Third, a systematic evaluation of both TyG_TWA and CHG_TWA was conducted, revealing that CHG_TWA had a modestly higher discriminatory ability for CVD compared with TyG_TWA. Lastly, this study provided a comprehensive assessment of the associations between TyG_TWA and CHG_TWA and CVD risk across various stages of CKM syndrome, aligning with the AHA statement emphasizing early monitoring and intervention in CKM syndrome. Nevertheless, the limitations of this study should be acknowledged. First, direct measurements of subclinical CVD, including coronary artery calcification, were unavailable in this study. Therefore, CKM syndrome stage 3 was defined based on subclinical CVD risk equivalents. Although this approach is consistent with current CKM stage criteria, it may introduce potential overlap between CKM classification, metabolic risk factors, and subsequent CVD risk. However, sensitivity analyses restricted to CKM stages 0–2 yielded consistent results, supporting the robustness of our findings. Second, although plasma N-terminal pro-B-type natriuretic peptide level was not collected, heart failure diagnoses were based on ICD codes from electronic medical records and confirmed by specialized cardiovascular physicians. Third, causal relationships cannot be established because this study is an observational study. Moreover, residual confounding cannot be entirely ruled out due to limitations in the initial study design, however, the concerns were mitigated as much as possible by adjusting for multiple covariates, ensuring a large sample size, and complete follow-up pathways. Although sensitivity analyses excluding participants receiving antihypertensive, hypoglycemic medications, or lipid-lowering medications yielded consistent results, the lack of detailed information on medication dosage, duration, and treatment response may have introduced residual confounding. Finally, the study population comprised Chinese individuals, and validation in other international cohorts is warranted.

## Conclusions

In conclusion, we demonstrated that elevated TyG_TWA and CHG_TWA were associated with an increased risk of CVD among populations with CKM syndrome stages 0-3, and this association was further strengthened with longer durations of exposure to high TyG index and high CHG index. The CHG index had a modestly higher discriminatory ability for CVD risk compared with the TyG index. In the context of CKM syndrome, longitudinal assessment of TyG index and CHG index may provide additional information for CVD risk stratification and facilitate early identification of individuals at higher risk of CVD.

## Data Availability

The datasets used and/or analyzed during the current study are available from the corresponding author on reasonable request. Requests to access these datasets should be directed to Yuntao Wu wyt0086@163.com.

## References

[1] Ndumele CE Rangaswami J Chow SL Neeland IJ Tuttle KR Khan SS . Cardiovascular-kidney-metabolic health: A presidential advisory from the American Heart Association. Circulation. (2023) 148:1606–35. doi: 10.1161/cir.0000000000001184 37807924

[2] Ndumele CE Neeland IJ Tuttle KR Chow SL Mathew RO Khan SS . A synopsis of the evidence for the science and clinical management of cardiovascular-kidney-metabolic (CKM) syndrome: A scientific statement from the American Heart Association. Circulation. (2023) 148:1636–64. doi: 10.1161/cir.0000000000001186 37807920

[3] Kadowaki T Maegawa H Watada H Yabe D Node K Murohara T . Interconnection between cardiovascular, renal and metabolic disorders: A narrative review with a focus on Japan. Diabetes Obes Metab. (2022) 24:2283–96. doi: 10.1111/dom.14829 35929483 PMC9804928

[4] Xie P Wang Y Xu X Tan L Guo X Xu Z . Association of cardiovascular-kidney-metabolic syndrome with cognitive decline and dementia: The ARIC study insights. J Am Heart Assoc. (2025) 14:e038445. doi: 10.1161/JAHA.124.038445 PMC12184565 40371582

[5] Liu X Zhang H Li H Xue F . Cardiovascular-kidney-metabolic syndrome modifies smoking-related risk for cardiovascular diseases: Findings from an observational cohort study in UK Biobank. BMC Public Health. (2025) 25:1609. doi: 10.1186/s12889-025-22865-3 40312716 PMC12044822

[6] Roth S M'Pembele R Matute P Kotfis K Larmann J Lurati Buse G . Cardiovascular-kidney-metabolic syndrome: Association with adverse events after major noncardiac surgery. Anesth Analg. (2024) 139:679–81. doi: 10.1213/ane.0000000000006975 39159243

[7] Zheng C Cai A Sun M Wang X Song Q Pei X . Prevalence and mortality of cardiovascular-kidney-metabolic syndrome in China: A nationwide population-based study. JACC Asia. (2025) 5:898–910. doi: 10.1016/j.jacasi.2025.04.007 40498431 PMC12277190

[8] Aggarwal R Ostrominski JW Vaduganathan M . Prevalence of cardiovascular-kidney-metabolic syndrome stages in US adults, 2011-2020. Jama. (2024) 331:1858–60. doi: 10.1001/jama.2024.6892 38717747 PMC11079779

[9] Tao LC Xu JN Wang TT Hua F Li JJ . Triglyceride-glucose index as a marker in cardiovascular diseases: Landscape and limitations. Cardiovasc Diabetol. (2022) 21:68. doi: 10.1186/s12933-022-01511-x 35524263 PMC9078015

[10] Liu X Tan Z Huang Y Zhao H Liu M Yu P . Relationship between the triglyceride-glucose index and risk of cardiovascular diseases and mortality in the general population: A systematic review and meta-analysis. Cardiovasc Diabetol. (2022) 21:124. doi: 10.1186/s12933-022-01546-0 35778731 PMC9250255

[11] Mansoori A Nosrati M Dorchin M Mohammadyari F Derakhshan-Nezhad E Ferns G . A novel index for diagnosis of type 2 diabetes mellitus: Cholesterol, high density lipoprotein, and glucose (CHG) index. J Diabetes Investig. (2025) 16:309–14. doi: 10.1111/jdi.14343 39569998 PMC11786182

[12] Mo D Zhang P Zhang M Dai H Guan J . Cholesterol, high-density lipoprotein, and glucose index versus triglyceride-glucose index in predicting cardiovascular disease risk: A cohort study. Cardiovasc Diabetol. (2025) 24:116. doi: 10.1186/s12933-025-02675-y 40065297 PMC11895360

[13] Tian X Wang A Wu S Zuo Y Chen S Zhang L . Cumulative serum uric acid and its time course are associated with risk of myocardial infarction and all-cause mortality. J Am Heart Assoc. (2021) 10:e020180. doi: 10.1161/jaha.120.020180 34120449 PMC8403320

[14] Li C Zhu Y Ma Y Hua R Zhong B Xie W . Association of cumulative blood pressure with cognitive decline, dementia, and mortality. J Am Coll Cardiol. (2022) 79:1321–35. doi: 10.1016/j.jacc.2022.01.045 35393012

[15] Wu S An S Li W Lichtenstein AH Gao J Kris-Etherton PM . Association of trajectory of cardiovascular health score and incident cardiovascular disease. JAMA Netw Open. (2019) 2:e194758. doi: 10.1001/jamanetworkopen.2019.4758 31150075 PMC6547110

[16] Huo Z Huang Z Feng J Li J Chen S Wang G . Life's Essential 8 and heart failure among patients with chronic kidney disease: The Kailuan Cohort Study. Eur J Prev Cardiol. (2024) 31:824–31. doi: 10.1093/eurjpc/zwad398 38113400

[17] Jin C Chen S Vaidya A Wu Y Wu Z Hu FB . Longitudinal change in fasting blood glucose and myocardial infarction risk in a population without diabetes. Diabetes Care. (2017) 40:1565–72. doi: 10.2337/dc17-0610 28887409 PMC5652588

[18] Levey AS Stevens LA Schmid CH Zhang YL Castro A Feldman HI . A new equation to estimate glomerular filtration rate. Ann Intern Med. (2009) 150:604–12. doi: 10.7326/0003-4819-150-9-200905050-00006 19414839 PMC2763564

[19] Simental-Mendía LE Rodríguez-Morán M Guerrero-Romero F . The product of fasting glucose and triglycerides as surrogate for identifying insulin resistance in apparently healthy subjects. Metab Syndr Relat Disord. (2008) 6:299–304. doi: 10.1089/met.2008.0034 19067533

[20] Zhang Y Chen S Tian X Wang P Xu Q Xia X . Association between cumulative atherogenic index of plasma exposure and risk of myocardial infarction in the general population. Cardiovasc Diabetol. (2023) 22:210. doi: 10.1186/s12933-023-01936-y 37592247 PMC10436658

[21] World Health Organization . International Statistical Classification of Diseases and Related Health Problems, 10th Revision. Geneva: World Health Organization (2010).

[22] Tunstall-Pedoe H Kuulasmaa K Amouyel P Arveiler D Rajakangas AM Pajak A . Myocardial infarction and coronary deaths in the World Health Organization MONICA Project. Registration procedures, event rates, and case-fatality rates in 38 populations from 21 countries in four continents. Circulation. (1994) 90:583–612. doi: 10.1161/01.cir.90.1.583 8026046

[23] Stroke--1989. Recommendations on stroke prevention, diagnosis, and therapy. Report of the WHO Task Force on Stroke and other Cerebrovascular Disorders. Stroke. (1989) 20:1407–31. doi: 10.1161/01.str.20.10.1407 2799873

[24] Heart Failure Group of Chinese Society of Cardiology of Chinese Medical Association, Chinese Heart Failure Association of Chinese Medical Doctor Association, Editorial Board of Chinese Journal of Cardiology . Chinese guidelines for the diagnosis and treatment of heart failure 2018. Zhonghua Xin Xue Guan Bing Za Zhi. (2018) 46:760–89. doi: 10.3760/cma.j.issn.0253-3758.2018.10.004 30369168

[25] Camm AJ Kirchhof P Lip GY Schotten U Savelieva I Ernst S . Guidelines for the management of atrial fibrillation: The Task Force for the Management of Atrial Fibrillation of the European Society of Cardiology (ESC). Eur Heart J. (2010) 31:2369–429. doi: 10.1093/eurheartj/ehq278 20802247

[26] Wu Z Jin C Vaidya A Jin W Huang Z Wu S . Longitudinal patterns of blood pressure, incident cardiovascular events, and all-cause mortality in normotensive diabetic people. Hypertension. (2016) 68:71–7. doi: 10.1161/hypertensionaha.116.07381 27217407 PMC4982467

[27] Wang JG . Chinese guidelines for the prevention and treatment of hypertension (2024 revision). J Geriatr Cardiol. (2025) 22:1–149. doi: 10.26599/1671-5411.2025.01.008 40151633 PMC11937835

[28] Fox CS Golden SH Anderson C Bray GA Burke LE de Boer IH . Update on prevention of cardiovascular disease in adults with type 2 diabetes mellitus in light of recent evidence: A scientific statement from the American Heart Association and the American Diabetes Association. Diabetes Care. (2015) 38:1777–803. doi: 10.2337/dci15-0012 26246459 PMC4876675

[29] Joint Committee on the Chinese Guidelines for Lipid Management . Chinese guidelines for lipid management (2023). Zhonghua Xin Xue Guan Bing Za Zhi. (2023) 51:221–55. doi: 10.3760/cma.j.cn112148-20230119-00038 36925135

[30] Stevens PE Levin A . Evaluation and management of chronic kidney disease: Synopsis of the kidney disease: Improving global outcomes 2012 clinical practice guideline. Ann Intern Med. (2013) 158:825–30. doi: 10.7326/0003-4819-158-11-201306040-00007 23732715

[31] Yang X Li J Hu D Chen J Li Y Huang J . Predicting the 10-year risks of atherosclerotic cardiovascular disease in Chinese population: The China-PAR Project (Prediction for ASCVD Risk in China). Circulation. (2016) 134:1430–40. doi: 10.1161/circulationaha.116.022367 27682885

[32] Ogłuszka M Orzechowska M Jędroszka D Witas P Bednarek AK . Evaluate Cutpoints: Adaptable continuous data distribution system for determining survival in Kaplan-Meier estimator. Comput Methods Programs BioMed. (2019) 177:133–9. doi: 10.1016/j.cmpb.2019.05.023 31319941

[33] Tian X Chen S Zhang Y Zhang X Xu Q Wang P . Time course of the triglyceride glucose index accumulation with the risk of cardiovascular disease and all-cause mortality. Cardiovasc Diabetol. (2022) 21:183. doi: 10.1186/s12933-022-01617-2 36100896 PMC9472367

[34] Cui H Liu Q Wu Y Cao L . Cumulative triglyceride-glucose index is a risk for CVD: A prospective cohort study. Cardiovasc Diabetol. (2022) 21:22. doi: 10.1186/s12933-022-01456-1 35144621 PMC8830002

[35] Che B Zhong C Zhang R Pu L Zhao T Zhang Y . Triglyceride-glucose index and triglyceride to high-density lipoprotein cholesterol ratio as potential cardiovascular disease risk factors: An analysis of UK Biobank data. Cardiovasc Diabetol. (2023) 22:34. doi: 10.1186/s12933-023-01762-2 36797706 PMC9936712

[36] Hong J Zhang R Tang H Wu S Chen Y Tan X . Comparison of triglyceride glucose index and modified triglyceride glucose indices in predicting cardiovascular diseases incidence among populations with cardiovascular-kidney-metabolic syndrome stages 0-3: A nationwide prospective cohort study. Cardiovasc Diabetol. (2025) 24:98. doi: 10.1186/s12933-025-02662-3 40022122 PMC11871812

[37] Liu K Hu J Huang Y He D Zhang J . Triglyceride-glucose-related indices and risk of cardiovascular disease and mortality in individuals with cardiovascular-kidney-metabolic (CKM) syndrome stages 0-3: A prospective cohort study of 282,920 participants in the UK Biobank. Cardiovasc Diabetol. (2025) 24:277. doi: 10.1186/s12933-025-02842-1 40640813 PMC12243434

[38] Li W Shen C Kong W Zhou X Fan H Zhang Y . Association between the triglyceride glucose-body mass index and future cardiovascular disease risk in a population with cardiovascular-kidney-metabolic syndrome stage 0-3: A nationwide prospective cohort study. Cardiovasc Diabetol. (2024) 23:292. doi: 10.1186/s12933-024-02352-6 39113004 PMC11308445

[39] Lu L Chen Y Liu B Li X Wang J Nie Z . Association between cumulative changes of the triglyceride glucose index and incidence of stroke in a population with cardiovascular-kidney-metabolic syndrome stage 0-3: A nationwide prospective cohort study. Cardiovasc Diabetol. (2025) 24:202. doi: 10.1186/s12933-025-02754-0 40355933 PMC12070779

[40] Root Howard F . Insulin resistance and bronze diabetes. N Engl J Med. (1929) 201:201–6. doi: 10.1056/nejm192908012010501

[41] Adeva-Andany MM Martínez-Rodríguez J González-Lucán M Fernández-Fernández C Castro-Quintela E . Insulin resistance is a cardiovascular risk factor in humans. Diabetes Metab Syndr. (2019) 13:1449–55. doi: 10.1016/j.jacbts.2018.04.003 31336505

[42] Packer M . Potentiation of insulin signaling contributes to heart failure in type 2 diabetes: A hypothesis supported by both mechanistic studies and clinical trials. JACC Basic Transl Sci. (2018) 3:415–9. doi: 10.1016/j.jacbts.2018.04.003 PMC6058949 30062227

[43] DeFronzo RA Tobin JD Andres R . Glucose clamp technique: A method for quantifying insulin secretion and resistance. Am J Physiol. (1979) 237:E214–223. doi: 10.1152/ajpendo.1979.237.3.E214 382871

[44] Matthews DR Hosker JP Rudenski AS Naylor BA Treacher DF Turner RC . Homeostasis model assessment: Insulin resistance and beta-cell function from fasting plasma glucose and insulin concentrations in man. Diabetologia. (1985) 28:412–9. doi: 10.1007/bf00280883 3899825

[45] Minh HV Tien HA Sinh CT Thang DC Chen CH Tay JC . Assessment of preferred methods to measure insulin resistance in Asian patients with hypertension. J Clin Hypertens (Greenwich). (2021) 23:529–37. doi: 10.1111/jch.14155 33415834 PMC8029536

[46] Sánchez-García A Rodríguez-Gutiérrez R Mancillas-Adame L González-Nava V Díaz González-Colmenero A Solis RC . Diagnostic accuracy of the triglyceride and glucose index for insulin resistance: A systematic review. Int J Endocrinol. (2020) 2020:4678526. doi: 10.1155/2020/4678526 PMC7085845 32256572

[47] Larkin H . Here's what to know about cardiovascular-kidney-metabolic syndrome, newly defined by the AHA. Jama. (2023) 330:2042–3. doi: 10.1001/jama.2023.22276 37938824

[48] Longo M Zatterale F Naderi J Parrillo L Formisano P Raciti GA . Adipose tissue dysfunction as determinant of obesity-associated metabolic complications. Int J Mol Sci. (2019) 20:2358. doi: 10.3390/ijms20092358 31085992 PMC6539070

[49] Hill MA Yang Y Zhang L Sun Z Jia G Parrish AR . Insulin resistance, cardiovascular stiffening and cardiovascular disease. Metabolism. (2021) 119:154766. doi: 10.1016/j.metabol.2021.154766 33766485

[50] Tudurí E Soriano S Almagro L Montanya E Alonso-Magdalena P Nadal Á . The pancreatic β-cell in ageing: Implications in age-related diabetes. Ageing Res Rev. (2022) 80:101674. doi: 10.1016/j.arr.2022.101674 35724861

[51] Seals DR Jablonski KL Donato AJ . Aging and vascular endothelial function in humans. Clin Sci (Lond). (2011) 120:357–75. doi: 10.1042/cs20100476 21244363 PMC3482987

[52] Xu X Wang B Ren C Hu J Greenberg DA Chen T . Age-related impairment of vascular structure and functions. Aging Dis. (2017) 8:590–610. doi: 10.14336/ad.2017.0430 28966804 PMC5614324

[53] Herzog MJ Müller P Lechner K Stiebler M Arndt P Kunz M . Arterial stiffness and vascular aging: mechanisms, prevention, and therapy. Signal Transduct Target Ther. (2025) 10:282. doi: 10.1038/s41392-025-02346-0 40887468 PMC12399776

[54] Ahmed B Rahman AA Lee S Malhotra R . The implications of aging on vascular health. Int J Mol Sci. (2024) 25:11188. doi: 10.3390/ijms252011188 39456971 PMC11508873

[55] Lee JH Jeon S Lee HS Lee JW . Trajectories of triglyceride-glucose index changes and their association with all-cause and cardiovascular mortality: a competing risk analysis. Cardiovasc Diabetol. (2024) 23:364. doi: 10.1186/s12933-024-02457-y 39407266 PMC11481394

