# Supplementary material for: Comparative associations of cumulative cholesterol-HDL-glucose index and triglyceride-glucose index with risk of cardiovascular disease in individuals with cardiovascular-kidney-metabolic syndrome stages 0-3: a prospective cohort study
Source: Front Endocrinol (Lausanne). 2026 Sep 18;17:1950124. doi: 10.3389/fendo.2026.1950124 (PMC13630701; doi:10.3389/fendo.2026.1950124)
Supplement: Supplementary file 1 [file SupplementaryFile1.docx]

**Supplement contents**

**Figure S1. Determining cut-off values of TyG_TWA and CHG_TWA with maxstat algorithm**

A1: Plot of standardized log-rank statistics for TyG_TWA; A2: Histogram of TyG_TWA; A3: Kaplan-Meier plot according to optimized cut-point of TyG_TWA; B1: Plot of standardized log-rank statistics for CHG_TWA; B2: Histogram of CHG_TWA; B3: Kaplan-Meier plot according to optimized cut-point of CHG_TWA. TyG, triglyceride-glucose; CHG, cholesterol, high-density lipoprotein, and glucose; TWA, time-weighted average.

**Figure S2. HR and 95%CI for the association of TyG_TWA with the risk of stroke, myocardial infarction, heart failure, and atrial fibrillation by using restricted cubic spline regression with 3 knots with placed at the 25th, 50th, and 75th percentiles**

A: TyG_TWA and the risk of stroke; B: TyG_TWA and the risk of myocardial infarction; C: TyG_TWA and the risk of heart failure; D: TyG_TWA and the risk of atrial fibrillation. The red lines indicate HR, the blue lines indicate 95% CI. CVD, cardiovascular disease; TyG, triglyceride-glucose; TWA, time-weighted average; HR, hazard ratio; CI, confidence interval; BMI, body mass index; hs-CRP, high-sensitive C-reactive protein; CKD, chronic kidney disease.

Models adjusted for age, gender, BMI categories, drinking status, smoking status, physical exercise, educational status, hs-CRP categories, CKD, hypertension, diabetes mellitus, and dyslipidemia.

**Figure S3. HR and 95%CI for the association of CHG_TWA with the risk of stroke, myocardial infarction, heart failure, and atrial fibrillation by using restricted cubic spline regression with 3 knots with placed at the 25th, 50th, and 75th percentiles**

A: CHG_TWA and the risk of stroke; B: CHG_TWA and the risk of myocardial infarction; C: CHG_TWA and the risk of heart failure; D: CHG_TWA and the risk of atrial fibrillation. The red lines indicate HR, the blue lines indicate 95% CI. CVD, cardiovascular disease; CHG, cholesterol, high-density lipoprotein, and glucose; TWA, time-weighted average; HR, hazard ratio; CI, confidence interval; BMI, body mass index; hs-CRP, high-sensitive C-reactive protein; CKD, chronic kidney disease.

Models adjusted for age, gender, BMI categories, drinking status, smoking status, physical exercise, educational status, hs-CRP categories, CKD, hypertension, diabetes mellitus, and dyslipidemia.

**Table S1. Association of TyG_TWA, CHG_TWA, exposure duration of high TyG index, and exposure duration of high CHG index with the risk of stroke among participants with CKM syndrome stage 0-3**

**Table S2. Association of TyG_TWA, CHG_TWA, exposure duration of high TyG index, and exposure duration of high CHG index with the risk of myocardial infarction among participants with CKM syndrome stage 0-3**

**Table S3. Association of TyG_TWA, CHG_TWA, exposure duration of high TyG index, and exposure duration of high CHG index with the risk of heart failure among participants with CKM syndrome stage 0-3**

**Table S4. Association of TyG_TWA, CHG_TWA, exposure duration of high TyG index, and exposure duration of high CHG index with the risk of atrial fibrillation among participants with CKM syndrome stage 0-3**

**Table S5. Association of per SD increment in TyG_TWA or CHG_TWA with the risk of CVD among participants with different CKM syndrome stage at 2006 survey**

**Table S6. Association of per SD increment in TyG_TWA or CHG_TWA with the risk of CVD among participants with different CKM syndrome stage at 2010 survey**

**Table S7. Predictive performances of TyG_TWA and CHG_TWA for CVD**

**Table S8. Sensitivity analysis for the association of per SD increment in TyG_TWA and CHG_TWA with the risk of CVD among participants with CKM syndrome stage 0-3**

**Table S9. Sensitivity analysis for the association of exposure duration of high TyG index with the risk of CVD among participants with CKM syndrome stage 0-3**

**Table S10. Sensitivity analysis for the association of exposure duration of high CHG index with the risk of CVD among participants with CKM syndrome stage 0-3**

**Table S11. Association of TyG_TWA, CHG_TWA, exposure duration of high TyG index, and exposure duration of high CHG index with the risk of CVD among participants with CKM syndrome stages 0-2**

**Figure S1. Determining cut-off values of TyG_TWA and CHG_TWA with maxstat algorithm**

A1: Plot of standardized log-rank statistics for TyG_TWA; A2: Histogram of TyG_TWA; A3: Kaplan-Meier plot according to optimized cut-point of TyG_TWA; B1: Plot of standardized log-rank statistics for CHG_TWA; B2: Histogram of CHG_TWA; B3: Kaplan-Meier plot according to optimized cut-point of CHG_TWA. TyG, triglyceride-glucose; CHG, cholesterol, high-density lipoprotein, and glucose; TWA, time-weighted average.

**Figure S2. HR and 95%CI for the association of TyG_TWA with the risk of stroke,** **myocardial infarction, heart failure, and atrial fibrillation by using restricted cubic spline regression with 3 knots with placed at the 25th, 50th, and 75th percentiles**

A: TyG_TWA and the risk of stroke; B: TyG_TWA and the risk of myocardial infarction; C: TyG_TWA and the risk of heart failure; D: TyG_TWA and the risk of atrial fibrillation. The red lines indicate HR, the blue lines indicate 95% CI. CVD, cardiovascular disease; TyG, triglyceride-glucose; TWA, time-weighted average; HR, hazard ratio; CI, confidence interval; BMI, body mass index; hs-CRP, high-sensitive C-reactive protein; CKD, chronic kidney disease.

Models adjusted for age, gender, BMI categories, drinking status, smoking status, physical exercise, educational status, hs-CRP categories, CKD, hypertension, diabetes mellitus, and dyslipidemia.

**Figure S3. HR and 95%CI for the association of CHG_TWA with the risk of stroke, myocardial infarction, heart failure, and atrial fibrillation by using restricted cubic spline regression with 3 knots with placed at the 25th, 50th, and 75th percentiles**

A: CHG_TWA and the risk of stroke; B: CHG_TWA and the risk of myocardial infarction; C: CHG_TWA and the risk of heart failure; D: CHG_TWA and the risk of atrial fibrillation. The red lines indicate HR, the blue lines indicate 95% CI. CVD, cardiovascular disease; CHG, cholesterol, high-density lipoprotein, and glucose; TWA, time-weighted average; HR, hazard ratio; CI, confidence interval; BMI, body mass index; hs-CRP, high-sensitive C-reactive protein; CKD, chronic kidney disease.

Models adjusted for age, gender, BMI categories, drinking status, smoking status, physical exercise, educational status, hs-CRP categories, CKD, hypertension, diabetes mellitus, and dyslipidemia.

**Table S1. Association of TyG_TWA, CHG_TWA, exposure duration of high TyG index, and exposure duration of high CHG index with the risk of stroke among participants with CKM syndrome stages 0-3**

|  | Case/Total | Incidence ratio | Model 1 | Model 2 | Model 3 | Model 4 |
| --- | --- | --- | --- | --- | --- | --- |
| **TyG_TWA** | | | | | | |
| Q1 | 527/11706 | 3.96 (3.64-4.32) | Reference | Reference | Reference | Reference |
| Q2 | 671/11707 | 5.06 (4.69-5.46) | 1.25 (1.11-1.40) | 1.22 (1.09-1.37) | 1.18 (1.05-1.33) | 1.17 (1.04 -1.31) |
| Q3 | 803/11707 | 6.09 (5.68-6.52) | 1.51 (1.35-1.68) | 1.45 (1.30-1.62) | 1.36 (1.21-1.52) | 1.29 (1.16-1.45) |
| Q4 | 1010/11707 | 7.77 (7.30-8.26) | 1.98 (1.78-2.20) | 1.87 (1.68-2.09) | 1.69 (1.51-1.88) | 1.47 (1.30-1.66) |
| P for trend | - | - | <0.05 | <0.05 | <0.05 | <0.05 |
| Per SD increment | 3011/46827 | 5.71 (5.51-5.92) | 1.31 (1.26-1.35) | 1.28 (1.24-1.33) | 1.23 (1.19-1.28) | 1.17 (1.13-1.23) |
| **Exposure duration of high TyG index** | | | | | | |
| 0 years | 721/14753 | 4.31 (4.00-4.63) | Reference | Reference | Reference | Reference |
| 2 years | 609/10442 | 5.15 (4.76-5.58) | 1.22 (1.09-1.36) | 1.19 (1.07-1.33) | 1.13 (1.02-1.26) | 1.11 (0.99-1.23) |
| 4 years | 659/9549 | 6.13 (5.68-6.61) | 1.46 (1.31-1.62) | 1.39 (1.25-1.55) | 1.30 (1.17-1.45) | 1.22 (1.09-1.36) |
| 6 years | 1022/12083 | 7.61 (7.16-8.09) | 1.78 (1.62-1.96) | 1.68 (1.52-1.86) | 1.52 (1.37-1.67) | 1.30 (1.16-1.46) |
| P for trend | - | - | <0.05 | <0.05 | <0.05 | <0.05 |
| **CHG_TWA** | | | | | | |
| Q1 | 558/11706 | 4.17 (3.84-4.53) | Reference | Reference | Reference | Reference |
| Q2 | 636/11707 | 4.75 (4.40-5.14) | 1.11 (0.99-1.25) | 1.08 (0.96-1.21) | 1.06 (0.95-1.19) | 1.05 (0.94-1.18) |
| Q3 | 750/11707 | 5.65 (5.26-6.07) | 1.27 (1.13-1.41) | 1.21 (1.08-1.35) | 1.17 (1.05-1.31) | 1.13 (1.01-1.26) |
| Q4 | 1067/11707 | 8.38 (7.90-8.90) | 1.69 (1.53-1.87) | 1.59 (1.43-1.77) | 1.48 (1.34-1.65) | 1.23 (1.09-1.38) |
| P for trend | - | - | <0.05 | <0.05 | <0.05 | <0.05 |
| Per SD increment | 3011/46827 | 5.71 (5.51-5.92) | 1.28 (1.24-1.32) | 1.26 (1.22-1.30) | 1.23 (1.19-1.27) | 1.15 (1.10-1.20) |
| **Exposure duration of high CHG index** | | | | | | |
| 0 years | 1588/30163 | 4.62 (4.39-4.85) | Reference | Reference | Reference | Reference |
| 2 years | 695/9932 | 6.24 (5.79-6.72) | 1.29 (1.18-1.41) | 1.25 (1.15-1.37) | 1.22 (1.11-1.33) | 1.16 (1.05-1.27) |
| 4 years | 416/4228 | 9.11 (8.27-10.02) | 1.68 (1.50-1.87) | 1.60 (1.44-1.79) | 1.51 (1.35-1.68) | 1.32 (1.16-1.49) |
| 6 years | 312/2504 | 11.86 (10.62-13.26) | 2.14 (1.89-2.41) | 2.03 (1.80-2.30) | 1.88 (1.66-2.13) | 1.46 (1.26-1.70) |
| P for trend | - | - | <0.05 | <0.05 | <0.05 | <0.05 |

Abbreviation: BMI, body mass index; FBG, fast blood glucose; HDL-C, high-density lipoprotein cholesterol; TC, total cholesterol; TG, triglyceride; hs-CRP, high-sensitive C-reactive protein; eGFR, estimated glomerular filtration rate; TyG, triglyceride-glucose; CHG, cholesterol, high-density lipoprotein, and glucose; TWA, time-weighted average; CKM, cardiovascular-kidney-metabolic; CKD, chronic kidney disease. Incidence ratio is per 1000 person-years (95% CI).

Model 1 adjusted for age and gender.

Model 2 adjusted for covariates in model 1 plus BMI categories, drinking status, smoking status, physical exercise, and educational status.

Model 3 adjusted for covariates in model 2 plus hs-CRP categories, CKD, and hypertension.

Model 4 adjusted for covariates in model 3 plus diabetes mellitus and dyslipidemia.

**Table S2. Association of TyG_TWA, CHG_TWA, exposure duration of high TyG index, and exposure duration of high CHG index with the risk of myocardial infarction among participants with CKM syndrome stages 0-3**

|  | Case/Total | Incidence ratio | Model 1 | Model 2 | Model 3 | Model 4 |
| --- | --- | --- | --- | --- | --- | --- |
| **TyG_TWA** | | | | | | |
| Q1 | 100/11706 | 0.74 (0.61-0.90) | Reference | Reference | Reference | Reference |
| Q2 | 143/11707 | 1.06 (0.90-1.25) | 1.39 (1.08-1.80) | 1.37 (1.06-1.78) | 1.34 (1.04-1.73) | 1.28 (0.99-1.66) |
| Q3 | 203/11707 | 1.51 (1.32-1.73) | 1.99 (1.57-2.53) | 1.95 (1.53-2.48) | 1.83 (1.44-2.34) | 1.62 (1.26-2.07) |
| Q4 | 256/11707 | 1.92 (1.70-2.17) | 2.62 (2.08-3.30) | 2.55 (2.00-3.23) | 2.28 (1.80-2.91) | 1.61 (1.23-2.10) |
| P for trend | - | - | <0.05 | <0.05 | <0.05 | <0.05 |
| Per SD increment | 702/46827 | 1.31 (1.21-1.41) | 1.38 (1.29-1.47) | 1.36 (1.27-1.46) | 1.31 (1.22-1.41) | 1.13 (1.03-1.23) |
| **Exposure duration of high TyG index** | | | | | | |
| 0 years | 135/14753 | 0.80 (0.67-0.94) | Reference | Reference | Reference | Reference |
| 2 years | 133/10442 | 1.11 (0.93-1.31) | 1.42 (1.12-1.81) | 1.40 (1.10-1.78) | 1.34 (1.05-1.71) | 1.27 (1.00-1.62) |
| 4 years | 158/9549 | 1.44 (1.23-1.68) | 1.86 (1.48-2.34) | 1.81 (1.44-2.29) | 1.70 (1.35-2.15) | 1.49 (1.17-1.89) |
| 6 years | 276/12083 | 2.01 (1.79-2.26) | 2.55 (2.07-3.14) | 2.49 (2.01-3.08) | 2.22 (1.79-2.76) | 1.63 (1.28-2.08) |
| P for trend | - | - | <0.05 | <0.05 | <0.05 | <0.05 |
| **CHG_TWA** | | | | | | |
| Q1 | 86/11706 | 0.63 (0.51-0.78) | Reference | Reference | Reference | Reference |
| Q2 | 139/11707 | 1.02 (0.87-1.21) | 1.56 (1.19-2.04) | 1.52 (1.16-2.00) | 1.50 (1.14-1.96) | 1.45 (1.11-1.90) |
| Q3 | 180/11707 | 1.34 (1.15-1.55) | 1.94 (1.50-2.51) | 1.88 (1.45-2.44) | 1.80 (1.39-2.34) | 1.65 (1.27-2.14) |
| Q4 | 297/11707 | 2.28 (2.03-2.55) | 2.94 (2.31-3.74) | 2.82 (2.20-3.60) | 2.59 (2.02-3.31) | 1.94 (1.48-2.54) |
| P for trend | - | - | <0.05 | <0.05 | <0.05 | <0.05 |
| Per SD increment | 702/46827 | 1.31 (1.21-1.41) | 1.42 (1.33-1.51) | 1.40 (1.31-1.49) | 1.35 (1.27-1.45) | 1.22 (1.12-1.33) |
| **Exposure duration of high CHG index** | | | | | | |
| 0 years | 305/30163 | 0.87 (0.78-0.98) | Reference | Reference | Reference | Reference |
| 2 years | 197/9932 | 1.74 (1.51-2.00) | 1.89 (1.58-2.26) | 1.85 (1.54-2.21) | 1.79 (1.49-2.14) | 1.58 (1.31-1.90) |
| 4 years | 122/4228 | 2.60 (2.18-3.10) | 2.47 (2.00-3.05) | 2.37 (1.92-2.93) | 2.19 (1.77-2.71) | 1.71 (1.35-2.17) |
| 6 years | 78/2504 | 2.86 (2.29-3.57) | 2.65 (2.07-3.41) | 2.54 (1.98-3.27) | 2.30 (1.79-2.96) | 1.56 (1.14-2.12) |
| P for trend | - | - | <0.05 | <0.05 | <0.05 | <0.05 |

Abbreviation: BMI, body mass index; FBG, fast blood glucose; HDL-C, high-density lipoprotein cholesterol; TC, total cholesterol; TG, triglyceride; hs-CRP, high-sensitive C-reactive protein; eGFR, estimated glomerular filtration rate; TyG, triglyceride-glucose; CHG, cholesterol, high-density lipoprotein, and glucose; TWA, time-weighted average; CKM, cardiovascular-kidney-metabolic; CKD, chronic kidney disease. Incidence ratio is per 1000 person-years (95% CI).

Model 1 adjusted for age and gender.

Model 2 adjusted for covariates in model 1 plus BMI categories, drinking status, smoking status, physical exercise, and educational status.

Model 3 adjusted for covariates in model 2 plus hs-CRP categories, CKD, and hypertension.

Model 4 adjusted for covariates in model 3 plus diabetes mellitus and dyslipidemia.

**Table S3. Association of TyG_TWA, CHG_TWA, exposure duration of high TyG index, and exposure duration of high CHG index with the risk of heart failure among participants with CKM syndrome stages 0-3**

|  | Case/Total | Incidence ratio | Model 1 | Model 2 | Model 3 | Model 4 |
| --- | --- | --- | --- | --- | --- | --- |
| **TyG_TWA** | | | | | | |
| Q1 | 179/11706 | 1.33 (1.15-1.55) | Reference | Reference | Reference | Reference |
| Q2 | 185/11707 | 1.38 (1.19-1.59) | 1.02 (0.83-1.25) | 0.98 (0.80-1.20) | 0.95 (0.77-1.17) | 0.91 (0.74-1.12) |
| Q3 | 232/11707 | 1.73 (1.52-1.97) | 1.32 (1.09-1.60) | 1.24 (1.01-1.51) | 1.16 (0.95-1.42) | 1.04 (0.85-1.27) |
| Q4 | 311/11707 | 2.34 (2.10-2.62) | 1.94 (1.61-2.33) | 1.77 (1.47-2.15) | 1.61 (1.33-1.95) | 1.15 (0.92-1.44) |
| P for trend | - | - | <0.05 | <0.05 | <0.05 | 0.12 |
| Per SD increment | 907/46827 | 1.69 (1.59-1.81) | 1.34 (1.26-1.42) | 1.31 (1.22-1.39) | 1.26 (1.18-1.34) | 1.09 (1.01-1.18) |
| **Exposure duration of high TyG index** | | | | | | |
| 0 years | 233/14753 | 1.38 (1.21-1.57) | Reference | Reference | Reference | Reference |
| 2 years | 158/10442 | 1.32 (1.13-1.54) | 1.03 (0.84-1.27) | 1.00 (0.81-1.22) | 0.96 (0.78-1.17) | 0.91 (0.74-1.11) |
| 4 years | 194/9549 | 1.77 (1.54-2.04) | 1.42 (1.18-1.72) | 1.34 (1.10-1.63) | 1.27 (1.04-1.54) | 1.10 (0.90-1.34) |
| 6 years | 322/12083 | 2.35 (2.11-2.62) | 1.84 (1.56-2.18) | 1.70 (1.43-2.03) | 1.55 (1.30-1.84) | 1.13 (0.92-1.38) |
| P for trend | - | - | <0.05 | <0.05 | <0.05 | 0.13 |
| **CHG_TWA** | | | | | | |
| Q1 | 161/11706 | 1.19 (1.02-1.39) | Reference | Reference | Reference | Reference |
| Q2 | 170/11707 | 1.25 (1.08-1.46) | 1.08 (0.87-1.33) | 1.03 (0.83-1.28) | 1.01 (0.81-1.25) | 0.99 (0.80-1.23) |
| Q3 | 206/11707 | 1.53 (1.34-1.75) | 1.24 (1.01-1.53) | 1.17 (0.95-1.44) | 1.12 (0.91-1.38) | 1.04 (0.84-1.29) |
| Q4 | 370/11707 | 2.84 (2.57-3.15) | 1.92 (1.59-2.31) | 1.76 (1.45-2.12) | 1.62 (1.34-1.96) | 1.14 (0.92-1.42) |
| P for trend | - | - | <0.05 | <0.05 | <0.05 | 0.18 |
| Per SD increment | 907/46827 | 1.69 (1.59-1.81) | 1.36 (1.28-1.43) | 1.32 (1.25-1.40) | 1.29 (1.22-1.37) | 1.12 (1.04-1.20) |
| **Exposure duration of high CHG index** | | | | | | |
| 0 years | 430/30163 | 1.23 (1.12-1.36) | Reference | Reference | Reference | Reference |
| 2 years | 228/9932 | 2.01 (1.77-2.29) | 1.60 (1.36-1.87) | 1.54 (1.31-1.81) | 1.49 (1.27-1.75) | 1.32 (1.11-1.56) |
| 4 years | 130/4228 | 2.77 (2.33-3.29) | 1.75 (1.44-2.13) | 1.64 (1.35-2.00) | 1.54 (1.26-1.88) | 1.14 (0.91-1.43) |
| 6 years | 119/2504 | 4.39 (3.67-5.25) | 2.65 (2.16-3.24) | 2.48 (2.02-3.04) | 2.29 (1.86-2.81) | 1.37 (1.05-1.78) |
| P for trend | - | - | <0.05 | <0.05 | <0.05 | <0.05 |

Abbreviation: BMI, body mass index; FBG, fast blood glucose; HDL-C, high-density lipoprotein cholesterol; TC, total cholesterol; TG, triglyceride; hs-CRP, high-sensitive C-reactive protein; eGFR, estimated glomerular filtration rate; TyG, triglyceride-glucose; CHG, cholesterol, high-density lipoprotein, and glucose; TWA, time-weighted average; CKM, cardiovascular-kidney-metabolic; CKD, chronic kidney disease. Incidence ratio is per 1000 person-years (95% CI).

Model 1 adjusted for age and gender.

Model 2 adjusted for covariates in model 1 plus BMI categories, drinking status, smoking status, physical exercise, and educational status.

Model 3 adjusted for covariates in model 2 plus hs-CRP categories, CKD, and hypertension.

Model 4 adjusted for covariates in model 3 plus diabetes mellitus and dyslipidemia.

**Table S4. Association of TyG_TWA, CHG_TWA, exposure duration of high TyG index, and exposure duration of high CHG index with the risk of atrial fibrillation among participants with CKM syndrome stages 0-3**

|  | Case/Total | Incidence ratio | Model 1 | Model 2 | Model 3 | Model 4 |
| --- | --- | --- | --- | --- | --- | --- |
| **TyG_TWA** | | | | | | |
| Q1 | 112/11706 | 0.83 (0.69-1.00) | Reference | Reference | Reference | Reference |
| Q2 | 103/11707 | 0.76 (0.63-0.93) | 0.90 (0.69-1.18) | 0.84 (0.64-1.10) | 0.83 (0.63-1.09) | 0.83 (0.63-1.09) |
| Q3 | 109/11707 | 0.81 (0.67-0.98) | 0.98 (0.75-1.28) | 0.88 (0.67-1.15) | 0.85 (0.65-1.11) | 0.85 (0.64-1.11) |
| Q4 | 111/11707 | 0.83 (0.69-1.00) | 1.08 (0.83-1.41) | 0.94 (0.71-1.23) | 0.89 (0.68-1.17) | 0.86 (0.63-1.18) |
| P for trend | - | - | 0.46 | 0.73 | 0.47 | 0.34 |
| Per SD increment | 435/46827 | 0.81 (0.74-0.89) | 1.04 (0.95-1.15) | 0.99 (0.89-1.10) | 0.97 (0.88-1.07) | 0.95 (0.84-1.07) |
| **Exposure duration of high TyG index** | | | | | | |
| 0 years | 139/14753 | 0.82 (0.69-0.97) | Reference | Reference | Reference | Reference |
| 2 years | 90/10442 | 0.75 (0.61-0.92) | 0.98 (0.75-1.27) | 0.92 (0.71-1.20) | 0.90 (0.69-1.18) | 0.90 (0.69-1.17) |
| 4 years | 91/9549 | 0.83 (0.67-1.02) | 1.10 (0.85-1.44) | 1.00 (0.76-1.30) | 0.97 (0.74-1.27) | 0.95 (0.72-1.25) |
| 6 years | 115/12083 | 0.83 (0.69-1.00) | 1.09 (0.85-1.39) | 0.96 (0.74-1.23) | 0.91 (0.70-1.18) | 0.88 (0.65-1.17) |
| P for trend | - | - | 0.40 | 0.84 | 0.56 | 0.44 |
| **CHG_TWA** | | | | | | |
| Q1 | 98/11706 | 0.72 (0.59-0.88) | Reference | Reference | Reference | Reference |
| Q2 | 92/11707 | 0.68 (0.55-0.83) | 0.95 (0.71-1.26) | 0.89 (0.67-1.18) | 0.88 (0.66-1.17) | 0.88 (0.66-1.18) |
| Q3 | 118/11707 | 0.87 (0.73-1.05) | 1.16 (0.89-1.51) | 1.05 (0.80-1.37) | 1.03 (0.78-1.34) | 1.04 (0.79-1.36) |
| Q4 | 127/11707 | 0.97 (0.81-1.15) | 1.08 (0.83-1.41) | 0.94 (0.72-1.24) | 0.91 (0.69-1.19) | 0.80 (0.59-1.09) |
| P for trend | - | - | 0.33 | 0.97 | 0.73 | 0.35 |
| Per SD increment | 435/46827 | 0.81 (0.74-0.89) | 1.05 (0.96-1.14) | 1.00 (0.91-1.10) | 0.99 (0.90-1.08) | 0.92 (0.83-1.03) |
| **Exposure duration of high CHG index** | | | | | | |
| 0 years | 253/30163 | 0.72 (0.64-0.82) | Reference | Reference | Reference | Reference |
| 2 years | 98/9932 | 0.86 (0.71-1.05) | 1.16 (0.92-1.46) | 1.10 (0.87-1.39) | 1.08 (0.85-1.37) | 1.06 (0.83-1.36) |
| 4 years | 53/4228 | 1.12 (0.86-1.47) | 1.22 (0.91-1.64) | 1.12 (0.83-1.50) | 1.08 (0.80-1.46) | 0.98 (0.70-1.37) |
| 6 years | 31/2504 | 1.13 (0.79-1.60) | 1.18 (0.81-1.71) | 1.07 (0.74-1.56) | 1.03 (0.71-1.50) | 0.82 (0.52-1.29) |
| P for trend | - | - | 0.13 | 0.44 | 0.63 | 0.59 |

Abbreviation: BMI, body mass index; FBG, fast blood glucose; HDL-C, high-density lipoprotein cholesterol; TC, total cholesterol; TG, triglyceride; hs-CRP, high-sensitive C-reactive protein; eGFR, estimated glomerular filtration rate; TyG, triglyceride-glucose; CHG, cholesterol, high-density lipoprotein, and glucose; TWA, time-weighted average; CKM, cardiovascular-kidney-metabolic; CKD, chronic kidney disease. Incidence ratio is per 1000 person-years (95% CI).

Model 1 adjusted for age and gender.

Model 2 adjusted for covariates in model 1 plus BMI categories, drinking status, smoking status, physical exercise, and educational status.

Model 3 adjusted for covariates in model 2 plus hs-CRP categories, CKD, and hypertension.

Model 4 adjusted for covariates in model 3 plus diabetes mellitus and dyslipidemia.

**Table S5. Association of per SD increment in TyG_TWA or CHG_TWA with the risk of CVD among participants with different CKM syndrome stages at 2006 survey**

|  | Case/Total | Incidence ratio | HR (95% CI) |
| --- | --- | --- | --- |
| **TyG_TWA** | | | |
| Stage 0 | 132/3567 | 3.17 (2.68-3.76) | 1.37 (1.04-1.80) |
| Stage 1 | 342/6731 | 4.38 (3.94-4.87) | 1.26 (1.07-1.50) |
| Stage 2 | 2743/29735 | 8.18 (7.88-8.49) | 1.10 (1.05-1.15) |
| Stage 3 | 1422/6794 | 22.27 (21.14-23.46) | 1.05 (0.99-1.13) |
| **CHG_TWA** | | | |
| Stage 0 | 132/3567 | 3.17 (2.68-3.76) | 1.15 (0.90-1.47) |
| Stage 1 | 342/6731 | 4.38 (3.94-4.87) | 1.08 (0.93-1.26) |
| Stage 2 | 2743/29735 | 8.18 (7.88-8.49) | 1.11 (1.06-1.16) |
| Stage 3 | 1422/6794 | 22.27 (21.14-23.46) | 1.07 (1.01-1.14) |

Abbreviation: CVD, cardiovascular disease; BMI, body mass index; hs-CRP, high-sensitive C-reactive protein; HR, hazard ratio; TyG, triglyceride-glucose; CHG, cholesterol, high-density lipoprotein, and glucose; TWA, time-weighted average; CKM, cardiovascular-kidney-metabolic; CKD, chronic kidney disease; CI, confidence interval. Incidence ratio is per 1000 person-years (95% CI). Model adjusted for age, gender, BMI categories, drinking status, smoking status, physical exercise, educational status, hs-CRP categories, CKD, hypertension, diabetes mellitus, and dyslipidemia.

**Table S6. Association of per SD increment in TyG_TWA or CHG_TWA with the risk of CVD among participants with different CKM syndrome stages at 2010 survey**

|  | Case/Total | Incidence ratio | HR (95% CI) |
| --- | --- | --- | --- |
| **TyG_TWA** | | | |
| Stage 0 | 81/2960 | 2.32 (1.87-2.89) | 1.59 (1.14-2.22) |
| Stage 1 | 280/6097 | 3.94 (3.51-4.43) | 1.35 (1.14-1.60) |
| Stage 2 | 2158/26946 | 7.01 (6.72-7.31) | 1.15 (1.10-1.21) |
| Stage 3 | 2120/10824 | 20.15 (19.31-21.03) | 1.05 (0.99-1.10) |
| **CHG_TWA** | | | |
| Stage 0 | 81/2960 | 2.32 (1.87-2.89) | 1.53 (1.11-2.10) |
| Stage 1 | 280/6097 | 3.94 (3.51-4.43) | 1.14 (0.97-1.34) |
| Stage 2 | 2158/26946 | 7.01 (6.72-7.31) | 1.13 (1.07-1.19) |
| Stage 3 | 2120/10824 | 20.15 (19.31-21.03) | 1.08 (1.03-1.14) |

Abbreviation: CVD, cardiovascular disease; BMI, body mass index; hs-CRP, high-sensitive C-reactive protein; HR, hazard ratio; TyG, triglyceride-glucose; CHG, cholesterol, high-density lipoprotein, and glucose; TWA, time-weighted average; CKM, cardiovascular-kidney-metabolic; CKD, chronic kidney disease; CI, confidence interval. Incidence ratio is per 1000 person-years (95% CI). Model adjusted for age, gender, BMI categories, drinking status, smoking status, physical exercise, educational status, hs-CRP categories, CKD, hypertension, diabetes mellitus, and dyslipidemia.

**Table S7. Predictive performances of TyG_TWA and CHG_TWA for CVD**

| Variables | AUC (95% CI) | Cut-off | Sensitivity and specificity, % | Youden index | P for comparison |
| --- | --- | --- | --- | --- | --- |
| TyG_TWA | 0.576 (0.567-0.584) | 8.652 | 0.574/0.541 | 0.115 | Reference |
| CHG_TWA | 0.592 (0.583-0.601) | 5.157 | 0.465/0.673 | 0.139 | <0.001 |

Abbreviation: TyG, triglyceride-glucose; CHG, cholesterol, high-density lipoprotein, and glucose; TWA, time-weighted average; CVD, cardiovascular disease; AUC, area under the curve; CI, confidence interval.

**Table S8. Sensitivity analysis for the association of per SD increment in TyG_TWA and CHG_TWA with the risk of CVD among participants with CKM syndrome stages 0-3**

|  | **TyG_TWA** |  | **CHG_TWA** |
| --- | --- | --- | --- |
| **Sensitivity analysis 1** | | | |
| Incidence ratio | 7.92 (7.68-8.16) |  | 7.92 (7.68-8.16) |
| HR (95% CI) | 1.14 (1.10-1.19) |  | 1.12 (1.08-1.16) |
| **Sensitivity analysis 2** | | | |
| Incidence ratio | 7.54 (7.29-7.81) |  | 7.54 (7.29-7.81) |
| HR (95% CI) | 1.18 (1.13-1.23) |  | 1.14 (1.10-1.19) |
| **Sensitivity analysis 3** | | | |
| Incidence ratio | 8.92 (8.66-9.18) |  | 8.92 (8.66-9.18) |
| HR (95% CI) | 1.14 (1.10-1.18) |  | 1.13 (1.09-1.17) |
| **Sensitivity analysis 4** | | | |
| Incidence ratio | 8.94 (8.69-9.20) |  | 8.94 (8.69-9.20) |
| HR (95% CI) | 1.14 (1.10-1.18) |  | 1.13 (1.09-1.17) |

Abbreviation: CVD, cardiovascular disease; BMI, body mass index; hs-CRP, high-sensitive C-reactive protein; TyG, triglyceride-glucose; CHG, cholesterol, high-density lipoprotein, and glucose; TWA, time-weighted average; CKD, chronic kidney disease; CKM, cardiovascular-kidney-metabolic. Models adjusted for age, gender, BMI categories, drinking status, smoking status, physical exercise, and educational status, hs-CRP categories, CKD, hypertension, diabetes mellitus, and dyslipidemia. Incidence ratio is per 1000 person-years (95% CI).

Sensitivity analysis 1: exclusion of participants with incident cardiovascular disease within 2 years;

Sensitivity analysis 2: exclusion of participants with antihypertensive medications, hypoglycemic medications, or lipid-lowering medications;

Sensitivity analysis 3: exclusion of participants with history of cancer;

Sensitivity analysis 4: sensitivity analysis used competing risk model, considering death as a competing risk.

**Table S9. Sensitivity analysis for the association of exposure duration of high TyG index with the risk of CVD among participants with CKM syndrome stages 0-3**

|  | **Exposure duration of high TyG index** | | | | P for trend |
| --- | --- | --- | --- | --- | --- |
|  | 0 years | 2 years | 4 years | 6 years |  |
| **Sensitivity analysis 1 (N=45906)** | | | | | |
| Incidence ratio | 6.09 (5.72-6.47) | 7.10 (6.63-7.60) | 8.32 (7.78-8.88) | 10.63 (10.09-11.21) | - |
| HR (95% CI) | Reference | 1.09 (1.00-1.20) | 1.18 (1.07-1.30) | 1.28 (1.17-1.41) | <0.05 |
| **Sensitivity analysis 2 (N=38231)** | | | | | |
| Incidence ratio | 6.03 (5.65-6.44) | 6.93 (6.43-7.47) | 8.07 (7.49-8.70) | 10.14 (9.51-10.80) | **-** |
| HR (95% CI) | Reference | 1.11 (1.01-1.23) | 1.23 (1.11-1.36) | 1.35 (1.21-1.50) | <0.05 |
| **Sensitivity analysis 3 (N=46520)** | | | | | |
| Incidence ratio | 6.79 (6.40-7.20) | 7.94 (7.44-8.47) | 9.50 (8.93-10.11) | 12.01 (11.43-12.62) | - |
| HR (95% CI) | Reference | 1.09 (1.01-1.19) | 1.20 (1.10-1.32) | 1.27 (1.16-1.39) | <0.05 |
| **Sensitivity analysis 4 (N=46827)** | | | | | |
| Incidence ratio | 6.81 (6.42-7.22) | 7.93 (7.44-8.46) | 9.53 (8.96-10.14) | 12.04 (11.46-12.65) | - |
| HR (95% CI) | Reference | 1.09 (1.00-1.19) | 1.21 (1.10-1.32) | 1.28 (1.16-1.40) | <0.05 |

Abbreviation: CVD, cardiovascular disease; BMI, body mass index; hs-CRP, high-sensitive C-reactive protein; TyG, triglyceride-glucose; CKD, chronic kidney disease; CKM, cardiovascular-kidney-metabolic. Models adjusted for age, gender, BMI categories, drinking status, smoking status, physical exercise, and educational status, hs-CRP categories, CKD, hypertension, diabetes mellitus, and dyslipidemia. Incidence ratio is per 1000 person-years (95% CI).

Sensitivity analysis 1: exclusion of participants with incident cardiovascular disease within 2 years;

Sensitivity analysis 2: exclusion of participants with antihypertensive medications, hypoglycemic medications, or lipid-lowering medications;

Sensitivity analysis 3: exclusion of participants with history of cancer;

Sensitivity analysis 4: sensitivity analysis used competing risk model, considering death as a competing risk.

**Table S10. Sensitivity analysis for the association of exposure duration of high CHG index with the risk of CVD among participants with CKM syndrome stages 0-3**

|  | **Exposure duration of high CHG index** | | | | P for trend |
| --- | --- | --- | --- | --- | --- |
|  | 0 years | 2 years | 4 years | 6 years |  |
| **Sensitivity analysis 1 (N=45906)** | | | | | |
| Incidence ratio | 6.31 (6.04-6.58) | 8.94 (8.40-9.52) | 12.84 (11.83-13.94) | 16.51 (15.00-18.17) | - |
| HR (95% CI) | Reference | 1.20 (1.11-1.30) | 1.30 (1.17-1.44) | 1.40 (1.23-1.60) | <0.05 |
| **Sensitivity analysis 2 (N=38231)** | | | | | |
| Incidence ratio | 6.26 (5.99-6.56) | 8.64 (8.04-9.28) | 12.89 (11.69-14.21) | 16.28 (14.27-18.56) | - |
| HR (95% CI) | Reference | 1.21 (1.11-1.32) | 1.31 (1.16-1.48) | 1.46 (1.24-1.73) | <0.05 |
| **Sensitivity analysis 3 (N=46520)** | | | | | |
| Incidence ratio | 7.01 (6.73-7.30) | 10.11 (9.53-10.73) | 14.71 (13.62-15.89) | 19.24 (17.61-21.03) | - |
| HR (95% CI) | Reference | 1.22 (1.13-1.31) | 1.31 (1.19-1.44) | 1.42 (1.26-1.61) | <0.05 |
| **Sensitivity analysis 4 (N=46827)** | | | | | |
| Incidence ratio | 7.03 (6.75-7.32) | 10.15 (9.57-10.76) | 14.73 (13.65-15.91) | 19.15 (17.52-20.93) | - |
| HR (95% CI) | Reference | 1.22 (1.13-1.31) | 1.30 (1.18-1.44) | 1.41 (1.24-1.60) | <0.05 |

Abbreviation: CVD, cardiovascular disease; BMI, body mass index; hs-CRP, high-sensitive C-reactive protein; CHG, cholesterol, high-density lipoprotein, and glucose; CKD, chronic kidney disease; CKM, cardiovascular-kidney-metabolic. Models adjusted for age, gender, BMI categories, drinking status, smoking status, physical exercise, and educational status, hs-CRP categories, CKD, hypertension, diabetes mellitus, and dyslipidemia. Incidence ratio is per 1000 person-years (95% CI).

Sensitivity analysis 1: exclusion of participants with incident cardiovascular disease within 2 years;

Sensitivity analysis 2: exclusion of participants with antihypertensive medications, hypoglycemic medications, or lipid-lowering medications;

Sensitivity analysis 3: exclusion of participants with history of cancer;

Sensitivity analysis 4: sensitivity analysis used competing risk model, considering death as a competing risk.

**Table S11. Association of TyG_TWA, CHG_TWA, exposure duration of high TyG index, and exposure duration of high CHG index with the risk of CVD** **among participants with CKM syndrome stages 0-2**

|  | Case/Total | Incidence ratio | Model 1 | Model 2 | Model 3 | Model 4 |
| --- | --- | --- | --- | --- | --- | --- |
| **TyG_TWA** | | | | | | |
| Q1 | 363/8303 | 3.75 (3.39-4.16) | Reference | Reference | Reference | Reference |
| Q2 | 453/8303 | 4.70 (4.28-5.15) | 1.23 (1.07-1.41) | 1.20 (1.04-1.37) | 1.18 (1.02-1.35) | 1.16 (1.01-1.33) |
| Q3 | 581/8303 | 6.06 (5.59-6.58) | 1.61 (1.41-1.83) | 1.53 (1.34-1.75) | 1.48 (1.29-1.69) | 1.43 (1.25-1.63) |
| Q4 | 654/8303 | 6.86 (6.35-7.41) | 1.84 (1.62-2.10) | 1.72 (1.51-1.97) | 1.63 (1.42-1.86) | 1.44 (1.24-1.67) |
| P for trend | - | - | <0.05 | <0.05 | <0.05 | <0.05 |
| Per SD increment | 2051/33212 | 5.34 (5.11-5.57) | 1.29 (1.23-1.34) | 1.26 (1.21-1.32) | 1.24 (1.18-1.29) | 1.19 (1.13-1.25) |
| **Exposure duration of high TyG index** | | | | | | |
| 0 years | 541/11502 | 4.04 (3.71-4.40) | Reference | Reference | Reference | Reference |
| 2 years | 468/7780 | 5.20 (4.75-5.69) | 1.31 (1.16-1.49) | 1.28 (1.13-1.45) | 1.24 (1.10-1.41) | 1.22 (1.08-1.38) |
| 4 years | 451/6654 | 5.86 (5.35-6.43) | 1.47 (1.30-1.67) | 1.40 (1.23-1.59) | 1.35 (1.19-1.53) | 1.28 (1.13-1.46) |
| 6 years | 591/7276 | 7.08 (6.53-7.68) | 1.79 (1.59-2.01) | 1.68 (1.48-1.89) | 1.58 (1.40-1.78) | 1.40 (1.22-1.60) |
| P for trend | **-** | **-** | <0.05 | <0.05 | <0.05 | <0.05 |
| **CHG_TWA** | | | | | | |
| Q1 | 422/8303 | 4.37 (3.97-4.80) | Reference | Reference | Reference | Reference |
| Q2 | 468/8303 | 4.84 (4.42-5.30) | 1.09 (0.95-1.24) | 1.04 (0.91-1.19) | 1.04 (0.91-1.19) | 1.03 (0.90-1.17) |
| Q3 | 490/8303 | 5.08 (4.65-5.55) | 1.12 (0.98-1.27) | 1.05 (0.92-1.20) | 1.05 (0.92-1.19) | 1.01 (0.89-1.16) |
| Q4 | 671/8303 | 7.09 (6.57-7.65) | 1.50 (1.33-1.69) | 1.38 (1.22-1.56) | 1.37 (1.21-1.55) | 1.18 (1.03-1.35) |
| P for trend | - | - | <0.05 | <0.05 | <0.05 | <0.05 |
| Per SD increment | 2051/33212 | 5.34 (5.11-5.57) | 1.21 (1.16-1.27) | 1.18 (1.13-1.23) | 1.18 (1.13-1.23) | 1.10 (1.05-1.16) |
| **Exposure duration of high CHG index** | | | | | | |
| 0 years | 1259/23627 | 4.58 (4.34-4.84) | Reference | Reference | Reference | Reference |
| 2 years | 501/6651 | 6.56 (6.01-7.16) | 1.42 (1.28-1.58) | 1.37 (1.23-1.52) | 1.36 (1.22-1.51) | 1.29 (1.15-1.43) |
| 4 years | 187/2036 | 8.07 (6.99-9.31) | 1.63 (1.40-1.90) | 1.54 (1.32-1.80) | 1.51 (1.29-1.76) | 1.31 (1.11-1.55) |
| 6 years | 104/898 | 10.35 (8.54-12.55) | 2.12 (1.73-2.58) | 1.97 (1.61-2.41) | 1.98 (1.62-2.43) | 1.51 (1.20-1.90) |
| P for trend | - | - | <0.05 | <0.05 | <0.05 | <0.05 |

Abbreviation: CVD, cardiovascular disease; BMI, body mass index; FBG, fast blood glucose; HDL-C, high-density lipoprotein cholesterol; TC, total cholesterol; TG, triglyceride; hs-CRP, high-sensitive C-reactive protein; eGFR, estimated glomerular filtration rate; TyG, triglyceride-glucose; CHG, cholesterol, high-density lipoprotein, and glucose; TWA, time-weighted average; CKM, cardiovascular-kidney-metabolic; CKD, chronic kidney disease. Incidence ratio is per 1000 person-years (95% CI).

Model 1 adjusted for age and gender.

Model 2 adjusted for covariates in model 1 plus BMI categories, drinking status, smoking status, physical exercise, and educational status.

Model 3 adjusted for covariates in model 2 plus hs-CRP categories, CKD, and hypertension.

Model 4 adjusted for covariates in model 3 plus diabetes mellitus and dyslipidemia.
